# 4-Hexylresorcinol and silk sericin increase the expression of vascular endothelial growth factor via different pathways

**DOI:** 10.1038/s41598-019-40027-5

**Published:** 2019-03-05

**Authors:** You-Young Jo, Dae-Won Kim, Je-Yong Choi, Seong-Gon Kim

**Affiliations:** 1 Sericultural and Apicultural Division, National Institute of Agricultural Science, RDA, Wanju, 55365 Republic of Korea; 2 Department of Oral Biochemistry, College of Dentistry, Gangneung-Wonju National University, Gangneung, 28644 Republic of Korea; 3 School of Biochemistry and Cell Biology, BK21 Plus KNU Biomedical Convergence Program, Skeletal Diseases Analysis Center, Korea Mouse Phenotyping Center (KMPC), Kyungpook National University, Daegu, 41944 Republic of Korea; 4 Department of Oral and Maxillofacial Surgery, College of Dentistry, Gangneung-Wonju National University, Gangneung, 28644 Republic of Korea

## Abstract

Angiogenesis plays an important role in active inflammation and wound healing. Our results showed that silk sericin and 4-hexylresorcinol (4HR) increased vascular endothelial growth factor (VEGF) expression in a dose-dependent manner in RAW264.7 cells. Unlike 4HR, silk sericin increased the expression of hypoxia inducible factor-1α (HIF-1α) and HIF-2α. Pretreatment with an HIF inhibitor decreased the sericin-induced increase in VEGF expression. However, the HIF inhibitor did not affect the 4HR-induced increase in VEGF expression. An inhibitor of matrix metalloproteinase (MMP) declined the 4HR-induced increase in VEGF expression. Silk sericin increased production of reactive oxygen species (ROS), whereas 4HR decreased ROS. M1 markers were increased by silk sericin treatment, and M2 markers were increased by 4HR treatment. VEGF and angiogenin expression were higher in rats treated with a 4HR-incorporated silk mat than in rats treated with a silk mat alone. In conclusion, silk sericin and 4HR increased VEGF expression in RAW264.7 cells via HIF-mediated and MMP-mediated pathways, respectively. Silk sericin exerted like pro-oxidant effects and 4HR exerted anti-oxidant effects. Rats treated with a 4HR-incorporated silk mat showed higher levels of VEGF and angiogenin than those treated with a silk mat alone.

## Introduction

Angiogenesis is a vital step in wound healing. Development of pro-angiogenic drugs is necessary to promote wound healing in patients with ischemic disease. Many foreign materials such as bacterial toxins or alloplasts can increase the expression of pro-angiogenic genes at the wound site^1^. This type of angiogenesis is considered inflammation-induced angiogenesis^1^. Although the angiogenesis is an essential step in the healing process, the role of inflammation-induced angiogenesis is limited to uneventful wound healing. Inflammation-induced angiogenesis is a mechanism to counter infection and is required for increasing phagocytic activity^2^. Although inflammation is an important step in the course of wound healing, it should be a transient step^1,2^. Normal wound healing should be followed by resolution of inflammation^3^. Accordingly, a pro-angiogenic agent with rapid wound-healing and anti-inflammatory properties would be useful^1^.

Agents that induce dormancy in micro-organisms typically reduce the metabolic rate of the micro-organism as a pro-survival mechanism^4^. High metabolic rate results in increased the production of reactive oxygen species (ROS), which are typically genotoxic and associated with inflammation^5^. Considering the relationship between metabolic rate and ROS production, agents that induce dormancy in micro-organisms may reduce any activity that increases the metabolism, such as inflammation, in multi-cellular living organisms. The phase transition from the acute inflammation to the healing is poorly understood. Macrophages play an important role in this phase transition from inflammation to remodeling in wound healing^1,6^. Macrophages that induce active phagocytosis are known as “M1-like macrophages”, which generate high levels of ROS^6^. M2-like macrophages play an important role in the wound remodeling phase^7^. To date, no definite markers differentiate between M1 and M2 types of macrophages. ROS levels in macrophages are important in this phase transition.

Among various agents that induce dormancy in bacteria, resorcinol has been studied extensively^8^. 4-Hexylresorcinol (4HR) has been used as an antiseptics^9^ and anti-melanin agent in the food and cosmetic industries^10^. 4HR is a strong inducer of dormancy in micro-organisms^11^. It exerts anti-cancer effects due to pro-apoptotic activity^12,13^. 4HR inhibits the nuclear factor-kappa B (NF-kB) pathway^14,15^ and decreases expression of tumor necrosis factor-α (TNF-α) in macrophages^16^. Both NF-kB pathway and TNF-α play important roles in the inflammatory process^17^. Thus, 4HR can be used to accelerate the healing of deep burn wounds^16^. However, pro-angiogenic activity of 4HR has not been established. A recent study showed that 4HR increases levels of matrix metalloproteinases (MMPs) in the macrophages^18^. MMPs are proteolytic enzymes, and thus, the 4HR-induced increases in MMP expression accelerates the degradation of xenografts^19^ and silk fabric membranes^20^. The increase in pro-inflammatory MMPs induced by 4HR^18^ is contradictory to its anti-inflammatory effect^16^. 4HR has been reported to suppress foreign body giant cell formation^21^. MMP levels increase during the process of acute inflammation due to production by M1-like macrophages^6^. In addition, MMP levels increase during the tissue remodeling phase due to production by M2-like macrophages^7^. Levels of pro-angiogenic factors increase not only in chronic inflammation, but also under normal conditions. A membrane incorporated with silk and 4HR used for the guided bone regeneration technique was found to accelerate bone formation^22^. The anti-inflammatory effect of 4HR has been reported previously^16^. In this study, we investigated the pro-angiogenic effects of 4HR.

Silk sericin is a hydrophilic and adhesive protein produced by silkworms during formation of a cocoon^23^. In addition, silk sericin is an industrial byproducts that is considered a waste product^24^. The beneficial effects of silk sericin have been recently studied. Silk sericin is used in wound dressing materials and in cosmetics^24^. Silk sericin increases expression of TNF-α in a dose-dependent manner^23^. TNF-α is a strong activator of hypoxia inducible factors (HIFs)^25^, and HIFs can increase expression of vascular endothelial growth factors (VEGFs)^26^. Therefore, silk sericin may exert pro-angiogenic effects. Bacterial lipopolysaccharide (LPS) can increase the levels of HIFs and exert pro-angiogenic effects^27^. Silk sericin exhibits lower toxicity than LPS. However, the pro-angiogenic effects of silk sericin have not been examined.

As 4HR and silk sericin have different effects on expression of TNF-α in macrophages^16,23^, the mechanism underlying their pro-angiogenic effects are expected to be different. Regulation of expression of HIFs and VEGFs is important for uneventful wound healing. Expression of HIFs increases in certain wounds to prevent hypoxic damage and accelerate phagocytosis during the healing process. A pro-angiogenic drug that inhibits expression of TNF-α may be required for wounds that heal according to a HIF-independent mechanism of healing. The objective of this study was to evaluate the effects of 4HR and silk sericin on the expression of VEGF, HIF-1α, and HIF-2α. In addition, we used inhibitors of HIF and MMP to characterize the role of HIFs and MMPs in 4HR- or silk sericin-induced increases in VEGF expression.

## Results

### Sericin and 4HR increased expression of VEGF-A, VEGF-C, and angiogenin

**Figure 1:**
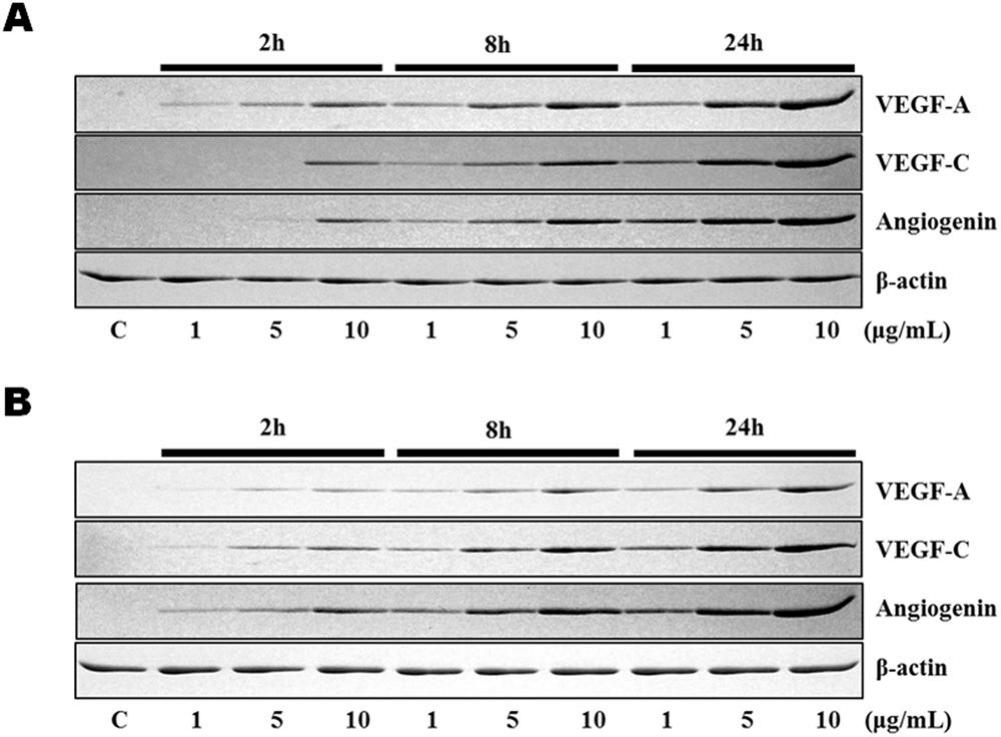
Expression of vascular endothelial growth factor A (VEGF-A), VEGF-C, and angiogenin after treatment with (**A**) sericin or (**B**) 4-hexylresorcinol (4HR). Sericin and 4HR increased expression of VEGF-A, VEGF-C, and angiogenin (cropped blot from different gels).

**Figure 2:**
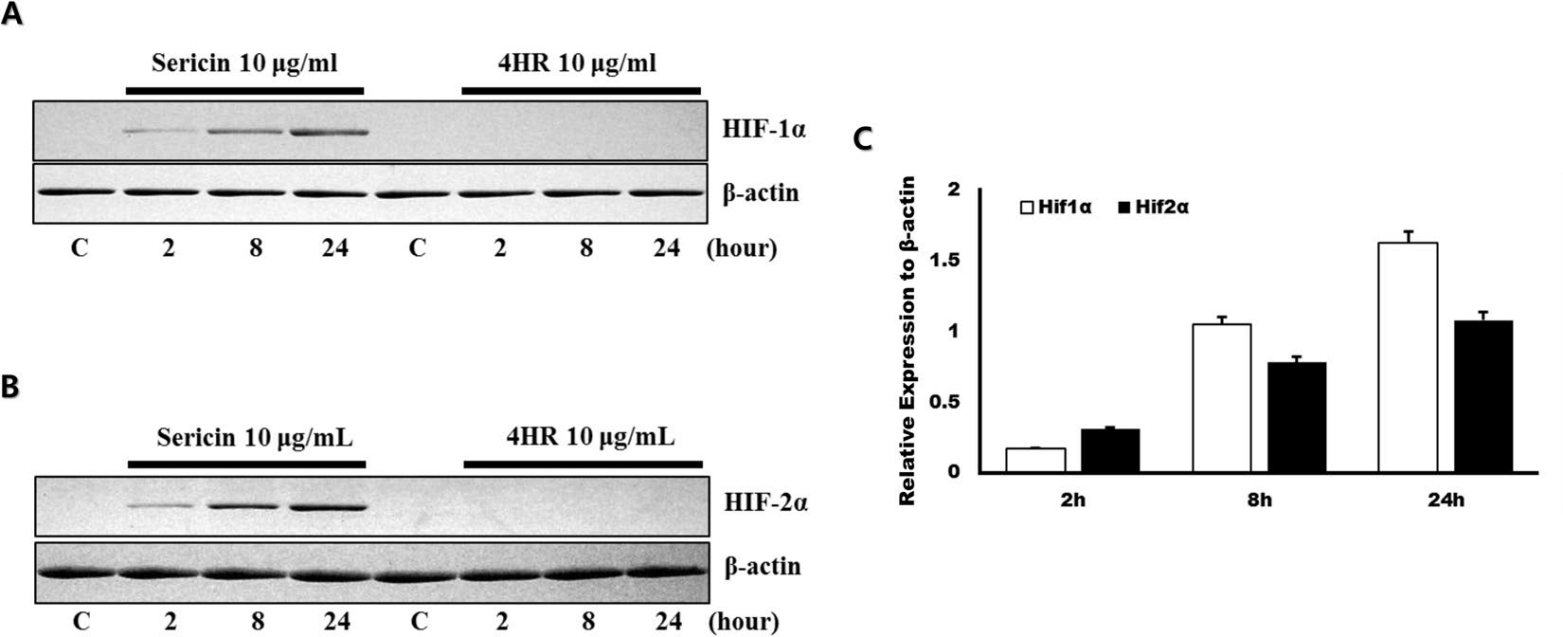
Expression of hypoxia inducible factor-1α (HIF-1α) and HIF-2α after treatment with (**A**) sericin or (**B**) 4-hexylresorcinol (4HR). Sericin increased expression of HIF-1α and HIF-2α. 4HR, at a dose equivalent to that of sericin, did not increase expression of HIF-1α or HIF-2α (cropped blot from different gels). (**C**) Relative expression of HIF-1α and HIF-2α to β-actin in sericin treated group. In case of 4HR group, expression levels of HIF-1α and HIF-2α was below the range of detection.

**Figure 3:**
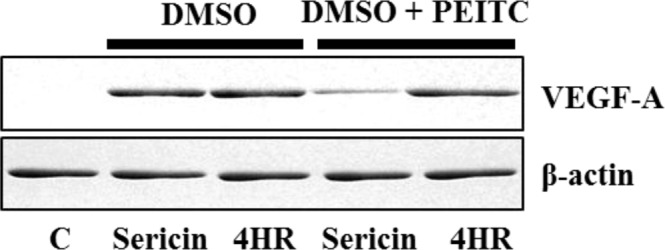
Expression of vascular endothelial growth factor A (VEGF-A) with or without pretreatment with the hypoxia inducible factor (HIF) inhibitor phenethyl isothiocyanate (PEITC). Pretreatment with PEITC decreased sericin-induced expression of VEGF-A. However, PEITC did not inhibit the 4-hexylresorcinol (4HR)-induced expression of VEGF-A (cropped blot from different gels).

Treatment of RAW264.7 cells with sericin or 4HR increased expression of VEGF-A, VEGF-C, and angiogenin in a dose-dependent manner (Fig. 1). As HIF is known to be a key transcription factor for angiogenesis^26^, the effect of sericin or 4HR administration on HIF expression was examined. Treatment with 10 μg/mL sericin, but not 4HR, increased the expression level of HIF-1α and HIF-2α (Fig. 2). To assess the relationship of HIF in the expression of VEGF-A, VEGF-C, and angiogenin, phenethyl isothiocyanate (PEITC), an inhibitor of HIF, was used. Pretreatment with PEITC decreased the sericin-induced, but not 4HR-induced, increase in VEGF-A, VEGF-C, and angiogenin expression (Fig. 3 and Supplementary Fig. 3B). Accordingly, sericin-induced increase in VEGF-A, VEGF-C, and angiogenin expression was HIF dependent.

**Figure 4:**
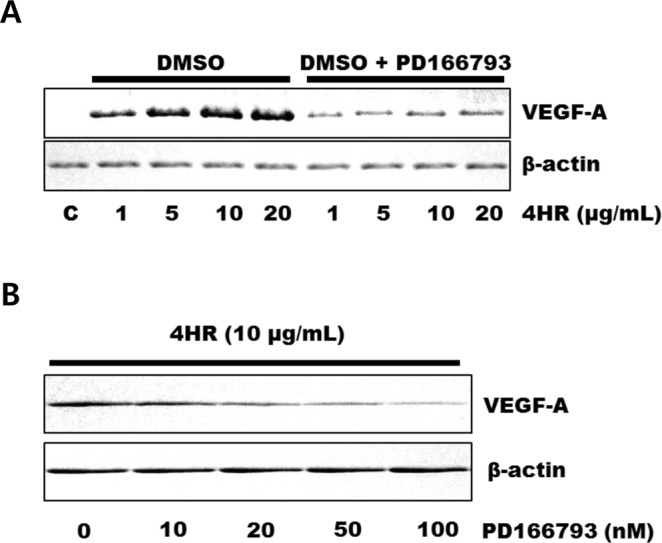
Expression of vascular endothelial growth factor A (VEGF-A) with or without pretreatment with the matrix metalloproteinase (MMP) inhibitor PD166793. (**A**) Pretreatment with PD166793 decreased 4-hexylresorcinol (4HR)-induced expression of VEGF-A (cropped blot from different gels). (**B**) According to pretreatment with PD166793, 10 μg/mL 4HR-induced expression of VEGF-A was inhibited as PD166793 dosage-dependent manner (cropped blot from different gels).

MMPs and VEGFs are expressed in both inflammatory phase and remodeling phase^1–3^. In addition, the expression of MMPs is closely associated with VEGF expression^28–30^. As 4HR increases the expression of MMP-2^18^, ARP100, an inhibitor of MMP-2, was used first. ARP100 did not decrease the 4HR-induced increase in VEGF-A expression (Supplementary Fig. 1). Accordingly, 4HR-induced increase in VEGF-A, VEGF-C, and angiogenin expression was not inhibited by blocking MMP-2 only. However, PD166793, broad-spectrum MMP inhibitor, decreased the 4HR-induced increase in VEGF-A, VEGF-C, and angiogenin expression (Fig. 4A and Supplementary Fig. 4B). According to the datasheet, the spectrum of MMP inhibition by PD166793 is dosage-dependent. As increasing PD166793 dosage, the inhibition spectrum of MMP is widening. When the applied 4HR concentration was set as 10 μg/mL, 4HR-induced VEGF-A expression was differentially inhibited by the dosage of PD166793 (Fig. 4B). As increasing the dosage of PD166793, 4HR-induced VEGF-A expression was inhibited more highly.

### Sericin increased ROS levels, but 4HR decreased ROS level

**Figure 5:**
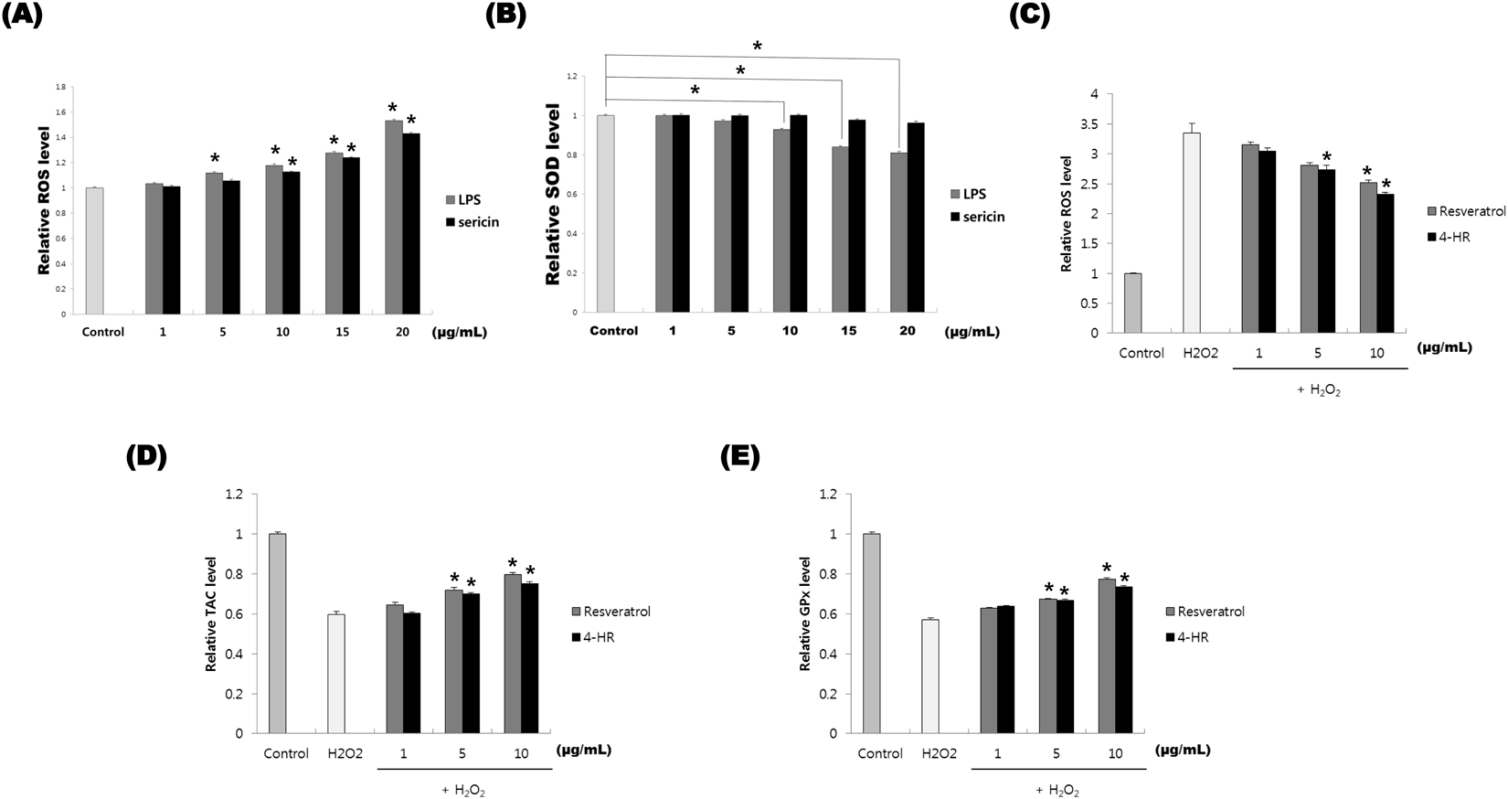
Sericin or 4-hexylresorcinol (4HR) administration and reactive oxygen species (ROS) level. (**A**) Treatment with lipopolysaccharide (LPS) or silk sericin both resulted in increased ROS levels. (**B**) Superoxide dismutase (SOD) activity. LPS treatment from 10 μg/mL to 20 μg/mL significantly increased SOD activity compared to that of controls (P < 0.005). Silk sericin treatment did not significantly alter SOD activity compared to that of controls. (P > 0.05). (**C**) ROS levels after 4HR or resveratrol treatment. H_2_O_2_ pre-treatment-induced ROS production was decreased after application of 4HR or resveratrol (*P < 0.05). (**D**) Total antioxidant capacity (TAC) after 4HR or resveratrol treatment. H_2_O_2_ pre-treatment-induced decreases in TAC were reversed by application of 4HR or resveratrol (*P < 0.05). (**E**) Glutathione peroxidase activity (GPx). Decreased GPx resulting from H_2_O_2_ pre-treatment was reversed by 4HR or resveratrol treatment (*P < 0.05).

The level of ROS is closely associated with inflammation^6^. M1 type macrophages generate high levels of ROS^6^. As M2-like macrophages are associated with remodeling, their ROS level is lower than that of M1-like macrophages^7^. The relative ROS levels in the untreated control were 0.781 ± 0.006 (Fig. 5A). LPS (5 μg/mL to 20 μg/mL, p < 0.001) and silk sericin (10 μg/mL to 20 μg/mL, p < 0.001) treatment resulted in increased ROS production. ROS levels were significantly higher in response to 20 µg/mL LPS compared to 20 µg/mL silk sericin (P < 0.001). LPS treatment from 10 μg/mL to 20 μg/mL resulted in significantly decreased SOD levels (Fig. 5B; P < 0.005), but silk sericin did not alter SOD levels (P > 0.05). The relative SOD levels in response to 10, 15, and 20 μg/mL LPS treatment were significantly lower than those in response to the corresponding concentrations of silk sericin (P < 0.005).

The relative ROS levels in the H_2_O_2_ treated control were 2.355 ± 0.272 (Fig. 5C). ROS levels were significantly different between the untreated control the 10 µg/mL resveratrol group (P = 0.001). Furthermore, 4HR significantly reduced ROS at 5 μg/mL and 10 μg/mL (P = 0.021 and P < 0.001, respectively). No differences were observed between the 4HR and resveratrol groups (P > 0.05).

The relative total antioxidant capacity in the H_2_O_2_ treated control was 0.336 ± 0.029 (Fig. 5D). Treatment with 5 μg/mL and 10 μg/mL resveratrol significantly increased total antioxidant capacity compared to that in controls (P = 0.012 and <0.001, respectively). In addition, treatment with 5 μg/mL and 10 μg/mL 4HR significantly increased antioxidant capacity compared to that of controls (P = 0.041 and 0.001, respectively). No differences were observed between the 4HR and resveratrol groups (P > 0.05).

The relative glutathione peroxidase activity in the H_2_O_2_ treated control was 0.275 ± 0.020 (Fig. 5E). Treatment with resveratrol significantly increased glutathione peroxidase activity at 5 μg/mL and 10 μg/mL (P = 0.003 and <0.001, respectively). 4HR significantly increased glutathione peroxidase activity at 5 μg/mL and 10 μg/mL (P = 0.005 and <0.001, respectively). No differences were observed between the resveratrol and 4HR groups (P > 0.05).

### Sericin increased M1 marker proteins, but 4HR M2 marker proteins

**Figure 6:**
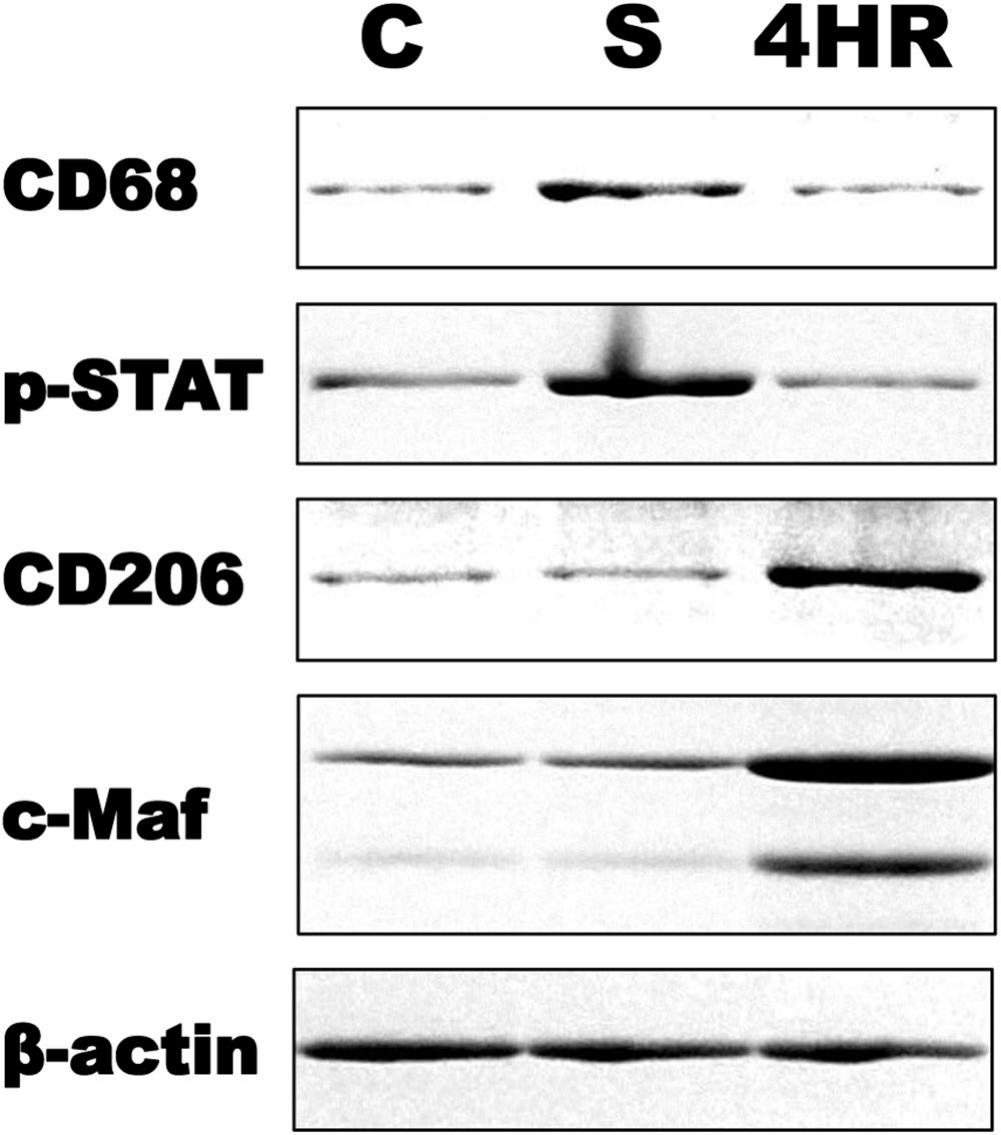
Sericin or 4-hexylresorcinol (4HR) administration and M1/M2 macrophage markers. Treatment with 10 μg/mL sericin increased expression of CD68 and pStat1. Treatment with 10 μg/mL 4HR increased expression of CD206 and c-Maf (C: untreated control).

M1 type macrophages play an important role in inflammation and M2 type macrophages play an important role in remodeling^1,6^. Treatment of RAW264.7 cells with 10 µg/mL sericin increased expression of the M1 markers CD68 and pStat1 (Fig. 6). Treatment of RAW264.7 cells with 10 μg/mL 4HR increased expression of the M2 markers CD206 and c-Maf (Fig. 6).

### Compared to a silk mat alone, a silk mat incorporated with 4HR increased the expression of VEGF-A, VEGF-C, and angiogenin

**Figure 7:**
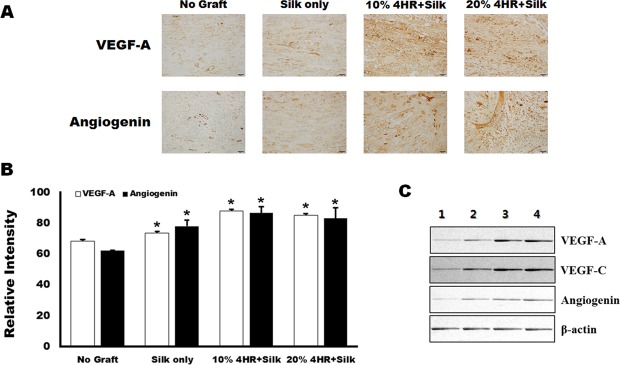
Silk mat with 4-hexylresorcinol (4HR) implantation animal model (n = 5 for each group). (**A**) Results of immunohistochemical analysis showed that expression of vascular endothelial growth factor (VEGF) and angiogenin was higher in the group treated with silk mats containing 4HR than in the group treated with silk mats alone. (**B**) VEGF-A and angiogenin expression were higher in both groups treated with silk mats with or without 4HR than in the control group (error bar = standard deviation, *P < 0.05). (**C**) Western blot from collected tissue samples. The results of western blotting were consistent with those of immunohistochemical analysis (cropped blot from different gels). Expression of VEGF-A, VEGF-C, and angiogenin increased in the group treated with silk mats containing 4HR (1: unfilled control, 2: silk mat alone, 3: silk mat with 10% 4HR, 4: silk mat with 20% 4HR).

Immunohistochemical analysis showed that the expression levels of VEGF-A and angiogenin were significantly higher in the group treated with the silk mat incorporated with 4HR than in the group treated with the silk mat alone (Fig. 7A,B). Furthermore, expression levels of VEGF-A and angiogenin were significantly higher in the group treated with the silk mat with or without 4HR than those in the group without any graft (P < 0.05). Western blot results were consistent with the results of immunohistochemical analysis (Fig. 7C). Although the expression level of VEGF-A, VEGF-C, and angiogenin were increased in the group exposed to the silk mat containing 4HR, no significant differences were observed in the expression levels between groups treated with the silk mats containing 10% and 20% 4HR (P > 0.05). Interestingly, von Willebrand factor (vWF) was also highly expressed in the endothelium following grafting with silk mats containing 10% and 20% 4HR. In groups treated with a silk mat alone or no graft, vWF were mostly observed in blood cells (Supplementary Fig. 5).

## Discussion

**Figure 8:**
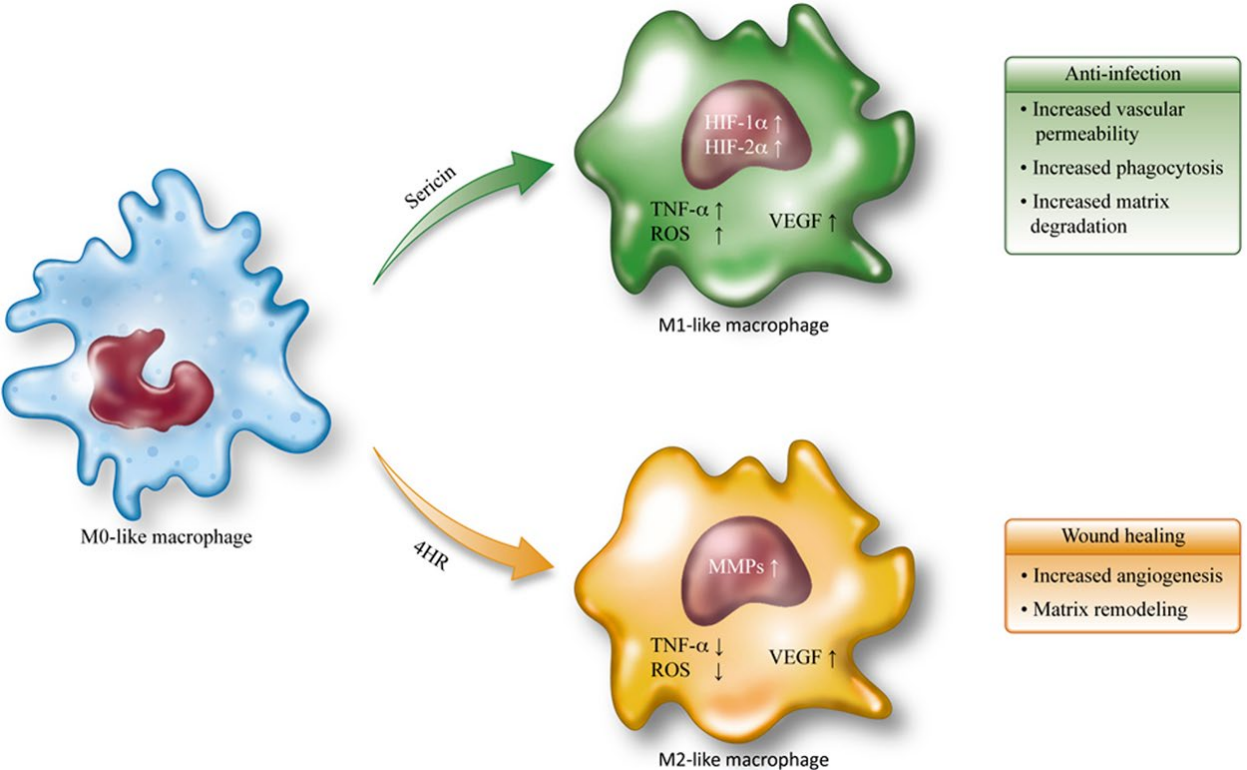
Sericin and 4-hexylresorcinol (4HR) increased expression of vascular endothelial growth factor (VEGF) via different mechanisms. Sericin increased expression of hypoxia inducible factor-1α (HIF-1α) and HIF-2α. These transcription factors increase expression of VEGFs and tumor necrosis factor-α (TNF-α). Increased expression of matrix metalloproteinases (MMPs) is an indirect consequence. These cytokines promote macrophages to increase their anti-infection ability. 4HR directly increased expression of MMPs but decreased expression of TNF-α and reduced production of reactive oxygen species (ROS). An increase in expression of MMPs results in increased expression of VEGFs. Thus, these effects promote wound healing via increased angiogenesis and matrix remodeling. The difference between M1-like macrophages and M2-like macrophages is small, and regulation in the cytokine expression determines macrophage polarization. Thus, treatment with sericin or 4HR may allow for intentional polarization of macrophages.

Our results showed that both sericin and 4HR increased expression of VEGF-A, VEGF-C, and angiogenin in *in vitro* (Fig. 1). In addition, expression levels of these proteins were increased *in vivo* in response to sericin and 4HR (Fig. 7). Sericin induced angiogenesis by increasing expression of HIF-1α and HIF-2α (Fig. 2). Sericin-induced increases in the expression of VEGF-A, VEGF-C, and angiogenin were inhibited by PEITC, which is a HIF inhibitor (Fig. 3). However, PEITC did not inhibit 4HR-induced expression of VEGF-A, VEGF-C, and angiogenin (Fig. 3 and Supplementary Fig. 3B). An MMP-2-selective inhibitor did not suppress the 4HR-induced increase in VEGF-A expression (Supplementary Fig. 1). A pan-MMP inhibitor (PD166793) decreased 4HR-induced VEGF-A, VEGF-C, and angiogenin expression (Fig. 4A and Supplementary Fig. 4B). ROS levels in RAW264.7 cells were increased by sericin administration, but were decreased by 4HR administration (Fig. 5). Expression level of M1 marker proteins was increased by sericin administration and expression of M2 marker proteins was increased by 4HR administration (Fig. 6). Thus, angiogenesis induced by 4HR was mediated by various MMPs and was independent of the HIF pathway (Fig. 8).

M1-like macrophages are associated with inflammation^6^, and many serious inflammatory diseases are associated with the stabilization of HIF^31^. M1-like macrophages induce active phagocytosis and they generate high levels of ROS^6^. M2-like macrophages play an important role in the wound remodeling phase^7^. Destruction of the vascular network by trauma, infection, or autoimmune attack can induce ischemia, and subsequent inflammation^31^. These conditions can stabilize HIF and increase the expression of VEGF^31^. M1 and M2 macrophages are characterized by different protein expression profiles, but most of these proteins are controversial as differential markers of macrophage polarization^32^. For examples, CD163+ cells have been traditionally identified as M2 macrophages^33,34^, but this characterization has been questioned recently^32^. CD206 was recently introduced as an M2 marker^35^. In this study, 4 different potential markers were used for identification of M1 and M2 macrophages (Fig. 6). Sericin increased the expression level of CD68 and pSTAT1 which are known to M1 markers^32^. CD206 expression was increased by the administration of 4HR (Fig. 6).

Sericin increased expression of TNF-α in a dose-dependent manner in our previous study^23^. Exposure to TNF-α increased HIF-1α expression in macrophages under normoxic conditions^36^. In this study, both sericin and LPS increased levels of ROS in RAW264.7 cells (Fig. 5A). SOD activity was decreased by sericin and LPS administration (Fig. 5B). Administration of sericin increased expression of HIF (Fig. 2). HIF is activated by ROS^37^, and a previous *in vitro* study showed that hydrogen peroxide stabilizes HIF-1α without inducing hypoxia^38^. Thus, activation of HIF by sericin administration might be mediated by increased ROS production. ROS are required for TNF-α-mediated stabilization of HIF-1α^39^. Furthermore, some molecules, such as LPS, increase the half-life of HIF via direct stabilization, and these molecules are typically pro-inflammatory^27^.

Considering the complex nature of the inflammatory response, sericin-induced increases in HIF expression may be beneficial or harmful depending on the specific type of HIF induced. In bacterial colitis, HIF-1α exerts protective effects on the mucosal barrier, but HIF-2α exerts pro-inflammatory effects^40^. In addition, HIF-1α plays an important role in macrophage function in infectious diseases^41^. AKB-4924 is a potent inducer of HIF-1α that exerts anti-microbial effects by activating macrophages^42^. Sericin has anti-microbial properties, but the underlying mechanisms have not been characterized. The anti-microbial effect of sericin may be partly mediated by the stabilization of HIF-1α (Fig. 8). Limb ischemia is a complication of diabetes mellitus, and administration of HIF-1α activators has been considered as a potential treatment^43^. However, HIF activators may increase risk of tumor growth^44^.

Our results showed that the HIF inhibitor, PEITC, inhibited sericin-induced, but not 4HR-induced, increase in VEGF expression (Fig. 3 and Supplementary Fig. 3B). Some antioxidants can inhibit HIF stabilization^45^. 4HR acts as an antioxidant by activating antioxidant enzymes^46^. In this study, 4HR decreased ROS levels in RAW264.7 cells previously treated with H_2_O_2_ (Fig. 5C). In addition, total antioxidant capacity in response to 4HR was similar to that of resveratrol (Fig. 5D). Therefore, our results showed that 4HR did not increase HIF expression (Fig. 2) and 4HR-induced VEGF expression was decreased by PD166793 (Fig. 4 and Supplementary Fig. 4B).

MMPs cause proteolysis of matrix components and induce release of VEGF from the matrix in tumors^28^. Some MMPs, such as MMP-9 and MMP-14, regulate VEGF expression, but the underlying mechanisms have not been characterized^29^. VEGF-A expression is positively correlated with expression of MMP-1, MMP-2, MMP-3, and MMP-9 in atherosclerotic lesions^30^. When the expression of MMP-2 and MMP-9 are inhibited by SB-3CT, selective MMP-2 and -9 inhibitor, VEGF-C expression is inhibited, too^47^. MMP-14 increases VEGF-C expression^48^. Angiogenin and MMP-9 are up-regulated in proangiogenic cells^49^. Treatment of murine macrophages with 4HR increased expression of MMP-2, MMP-3, and MMP-9^18^. Furthermore, administration of 4HR increased expression of MMP-13 and MMP-14 (Supplementary Fig. 2). An MMP-2 specific inhibitor (ARP100) did not decrease the 4HR-induced VEGF expression (Supplementary Fig. 1). PD166793 (20 nM) inhibited 4HR-induced expression of MMP-13 and VEGF (Fig. 4A). The IC_50_ of PD166793 for MMP-3 and MMP-13 were 12 nM and 8 nM, respectively^50^. Therefore, 20 nM of PD1666793 was sufficient to inhibit these MMPs. As increasing dosage of PD166793, inhibition of VEGF-A was increased (Fig. 4B). Inhibition of VEGF expression via MMP blockade has been observed in a xenograft model^51^. MMPs are mainly found in the extracellular matrix, but are also found in the nucleus^52^. MMP-3 and MMP-13 are often translocated to the nucleus and are involved in regulation of gene expression^53^. Nuclear MMPs may induce cellular apoptosis via proteolysis of nuclear proteins^54^, and may directly regulate gene transcription^55^. Intra-nuclear localization of MMPs and their involvement in transcription of VEGF might explain the mechanism of 4HR-induced VEGF expression (Fig. 8). However, how MMPs promoted VEGF expression in RAW264.7 cells remained unclear. To generalize our results, the effects of sericin and 4-HR on other macrophage cell lines or primary macrophages will be needed. These should be clarified forthcoming studies.

Therapeutic neovascularization is important in treatment of ischemic heart disease and diabetes mellitus, and in wound healing^56^. In a previous study, VEGF therapy increased neuronal survival in a model of cerebral ischemia^57^, and increased expression of VEGF facilitates coverage by pericytes and improves blood flow^58^. A previous study showed that use of a silk mat with 4HR for vessel repair promoted endothelial regeneration^59^. Thus, local delivery of VEGF may be beneficial in treating ischemia-induced tissue damage^60^. Our results showed that a silk mat incorporated with 4HR increased angiogenesis significantly more than a silk mat alone (Fig. 7). Silk sericin increased markers for M1-type macrophages and 4HR increased markers for M2-type macrophages (Fig. 6). M1- and M2-like macrophages play an important roles in uneventful wound healing^6,7^. Thus, our results indicate that a silk mat incorporated with 4HR may be used as a wound dressing or for the treatment of tissue damaged by ischemia.

## Conclusion

Silk sericin and 4HR increased VEGF expression in RAW264.7 cells via HIF-mediated and MMP-mediated pathways, respectively. Silk sericin increased ROS levels in RAW264.7 cells, while 4HR decreased ROS. Silk sericin increased M1 markers, while 4HR increased M2 markers in RAW264.7 cells. The group treated with silk mats containing 4HR showed higher VEGF and angiogenin expression than the group treated with the silk mat alone.

## Materials and Methods

### Cell cultures and 4HR/sericin treatment

RAW264.7 murine macrophages (Korean Cell Line Bank No. 40071) were suspended in culture medium^18,23^. Sericin was extracted by boiling silkworm cocoons kindly gifted by the Rural Development Administration (Wanju, Korea). 4HR was purchased from Sigma-Aldrich (St. Louis, MO, USA). RAW264.7 cells were placed in 6-well culture plates and treated with 1, 5, and 10 μg/mL of 4HR or sericin. After 2, 8, or 24 h of culture, the cells were collected. Cells in the control culture were treated with a volume of solvent equivalent to that required for 4HR and sericin.

### Western blotting

Proteins were collected and mixed with a sodium dodecyl sulfate buffer. After heat denaturation, they were electrophoresed on 10% polyacrylamide gels. The gels were transferred to polyvinylidene difluoride membranes. After blocking, the membranes were probed with primary antibodies (dilution ratio = 1:500). The sources and specifications of primary antibodies were listed in the Supplementary Data. Blots were imaged and quantified using a ChemiDoc XRS system (Bio-Rad Laboratories).

### Expression of HIFs and HIF-1α inhibition assay

To analyze expression of HIF-1α and HIF-2α induced by sericin or 4HR, we treated the cells with 10 μg/mL of 4HR or sericin. Subsequent steps for quantifying the different proteins were performed as described previously. The sources and specifications of primary antibodies were as follows: HIF-1α (Santa Cruz Biotech) and HIF-2α (Santa Cruz Biotech). PEITC is an inhibitor of HIF-1α^61^. The optimal concentration of PEITC was determined using hydrogen peroxide (known HIF-1α inducer) (Supplementary Fig. 3). RAW264.7 cells were pretreated with dimethyl sulfoxide (DMSO) or DMSO+T10 μM PEITC. Then, the cells were treated with 10 μg/mL of 4HR or sericin, cultured for 24 h, and subjected to western blot analysis for VEGF-A.

### Effects of MMPs inhibition on 4HR-induced VEGF expression

To analyze the effect of inhibition of MMPs on 4HR-induced VEGF expression, cells were treated with ARP100 or PD166793. ARP100 is an inhibitor of MMP-2^62^ and PD166793 is an inhibitor of various MMPs^50^. The optimal concentration of ARP100 or PD166793 was determined using an MMP-induction experiment (Supplementary Figs 1 and 4A). Our results showed that 30 nM ARP100 and 20 nM PD166793 inhibited MMP-2 and MMP-13, respectively. After treatment with the inhibitors, the cells were treated with 1, 5, 10, or 20 μg/mL of 4HR. Subsequently, we performed the experiment as described above. Cells were collected after 24 h of culture, and western blot analysis for VEGF-A was performed. To assess the influence of PD166793 dosage, 10, 20, 50, or 100 nM of PD166793 was used for the pretreatment. After treatment with different dosages of PD166793, the cells were treated with 10 μg/mL of 4HR. Subsequent procedure was in accord to the above.

### Measurement of ROS and total antioxidant capacity

ROS and superoxide levels after sericin administration were measured using commercial kits. The cellular culture conditions for RAW264.7 cells were the same as described above. Lipopolysaccharide (LPS) was used as positive control. Silk sericin or LPS were administered to RAW264.7 cells at 1, 5, 10, 15, and 20 μg/mL. After 24 h of cellular growth, ROS and superoxide levels were measured using a cellular ROS/Superoxide detection assay kit (CAT#: ab139476, Abcam). The procedure was performed in accordance with the manufacturer’ protocol. After incubating at 37 °C for 20 min, absorbance was measured at 450 nm. Superoxide dismutase activity was also evaluated under the same conditions using a commercial kit (CAT#: ab65354, Abcam).

Total antioxidant capacity after the administration of 4HR was measured using commercial kits. Cell culture conditions for RAW264.7 cells and H_2_O_2_ application conditions were the same as described above. Resveratrol was used as positive control. Resveratrol or 4HR were administered to RAW264.7 cells at 1, 5, and 10 μg/mL. After 24 h of cellular growth, total antioxidant capacity was measured using a total antioxidant capacity assay kit (CAT#: ab65329, Abcam). This procedure was performed according to the manufacturer’s protocol. After incubating at room temperature, absorbance was measured at 570 nm. Additionally, glutathione peroxidase assay was performed using a commercial kit (CAT#: ab102530, Abcam). After adding cumene hydroperoxide, absorbance was measured at 340 nm in a kinetic mode.

### Animals and experimental design

Eight-week-old Crl:CD (Sprague-Dawley) specific pathogen-free (SPF)/VAF outbred rats (Orientbio Inc., Sungnam, Korea) were used in this study. All procedures were performed in accordance with guidelines for laboratory animal care and were approved by the Gangneung-Wonju National University for animal research (GWNU-2017-17). Thirty six rats (2–3 rats per cage) were housed under a 12-h light/12-h dark cycle in a controlled environment at 20–22 °C and 40% humidity for one week for acclimation prior to experimentation. The rats had free access to food and water and were all fed a control semisynthetic diet according to a classical recommendation (74% carbohydrates from soybean vegetable oil, 14% proteins from casein, supplemented with a standard vitamin and mineral mix).

The rats were divided into four groups. Graft materials composed of silk, silk with 10% 4HR, and silk with 20% 4HR were divided into 0.2-g portions and sterilized using an autoclave. Group 1 (control) underwent anesthesia and an incision, but received no graft. Animals in group 2 were treated with a silk graft, animals in group 3 were treated with a silk graft containing 10% 4HR, and animals in group 4 were treated with a silk graft containing 20% 4HR. Prior to surgery, rats were anesthetized by intramuscular injection. The backs of the rats were shaved and disinfected using povidone-iodine, and a local anesthetic was applied. Subsequently, a vertical 1-cm incision was made from the arm pit position on the vertical midline deep enough to accommodate the grafts. The grafts were inserted into the subcutaneous layer and sutured using a 3–0 black silk suture. After four weeks, all rats were sacrificed, and histological and molecular biology analyses were performed.

### Immunohistochemical determination and western blot analysis in tissue samples

To assess the expression of VEGF-A and angiogenin, we performed immunohistochemical staining using anti-VEGF-A and anti-angiogenin antibodies (Santa Cruz Biotech). The procedure for immunohistochemical analysis was in accordance with a previous publication^18^. Briefly, the sections were prepared and enzyme predigestion was performed using a proteolytic enzyme (1 mg porcine trypsin, Sigma-Aldrich). Then, the sections were treated with hydrogen peroxide. After washing and blocking procedure, the sections were treated with primary antibodies (VEGF-A 1:50 and angiogenin 1:50). After conjugation with a universal secondary antibody (Dako REAL™ EnVision™/HRP, Rabbit/Mouse; Dako North America Inc.), the slides were stained with a mixture of diaminobenzidine chromogen and hydrogen peroxidase (Dako REAL™ DAB+ Chromogen and Dako REAL™ Substrate Buffer; Dako North America Inc). Evaluation of relative staining intensity was performed according to previous our previous publication^16^. Comparison among groups was done by ANOVA, with comparison between groups by Bonferroni’s method. Statistical significance was set at P < 0.05. In addition, immunoflurorescence staining was performed on tissue sections with a von Willebrand factor antibody (dilution ratio: 1:100). After application of FITC-conjugated secondary antibody, counterstaining was done with DAPI.

The tissues the surrounding the silk grafts were scraped from the backs of the rats, placed into micro-test tubes, and stored at −70 °C overnight (n = 5 for each group). The tissues were vigorously homogenized in a tissue protein extraction reagent buffer with a protease inhibitor cocktail, and western blot analysis was performed as previously reported^23^.

### Ethical approval and informed consent

The animal experiments in this study were approved by the Gangneung-Wonju National University for animal research (GWNU-2017-17). All animal experiments were performed in accordance with the relevant guidelines and regulations.

## Supplementary information

Supplementary information

## Data Availability

All datasets used in this study were provided as Supplementary Data.

## References

[1] Murad S (2014). Toll-like receptor 4 in inflammation and angiogenesis: a double-edged sword. Front. Immunol..

[2] Mahdavian Delavary B, van der Veer WM, van Egmond M, Niessen FB, Beelen RH (2011). Macrophages in skin injury and repair. Immunobiology.

[3] Swirski FK (2015). Inflammation and repair in the ischaemic myocardium. Hamostaseologie.

[4] Margulis AB, Il’inskaia ON, Kolpakov AI, El-Registran GI (2003). Induction of SOS-response of cells exposed to autoregulatory factors of microorganisms. Genetika.

[5] Filomeni G, De Zio D, Cecconi F (2015). Oxidative stress and autophagy: the clash between damage and metabolic needs. Cell Death Differ..

[6] Pinhal-Enfield G (2003). An angiogenic switch in macrophages involving synergy between Toll-like receptors 2, 4, 7, and 9 and adenosine A(2A) receptors. Am. J. Pathol..

[7] Wu WK, Llewellyn OP, Bates DO, Nicholson LB, Dick AD (2010). IL-10 regulation of macrophage VEGF production is dependent on macrophage polarisation and hypoxia. Immunobiology.

[8] Stasiuk M, Kozubek A (2010). Biological activity of phenolic lipids. Cell Mol. Life Sci..

[9] Evans RT, Baker PJ, Coburn RA, Fischman SL, Genco RJ (1977). *In vitro* antiplaque effects of antiseptic phenols. J. Periodontol..

[10] Rojas-Graü MA, Soliva-Fortuny R, Niartín-Belloso O (2008). Effect of natural antibrowning agents on color and related enzymes in fresh-cut Fuji apples as an alternative to the use of ascorbic acid. J. Food Sci..

[11] Kushneruk MA (2013). Factors inducing transition from growth to dormancy in rhizobacteria Azospirillum brasilense. Mikrobiologiia.

[12] Kim SG, Choi JY (2013). 4-hexylresorcinol exerts antitumor effects via suppression of calcium oscillation and its antitumor effects are inhibited by calcium channel blockers. Oncol. Rep..

[13] Lee SW (2013). Cisplatin and 4-hexylresorcinol synergise to decrease metastasis and increase survival rate in an oral mucosal melanoma xenograft model: a preliminary study. Tumour Biol..

[14] Kim SG, Lee SW, Park YW, Jeong JH, Choi JY (2011). 4-hexylresorcinol inhibits NF-κB phosphorylation and has a synergistic effect with cisplatin in KB cells. Oncol. Rep..

[15] Song JY, Kim SG, Park NR, Choi JY (2018). Porcine Bone Incorporated With 4-Hexylresorcinol Increases New Bone Formation by Suppression of the Nuclear Factor Kappa B Signaling Pathway. J. Craniofac. Surg..

[16] Ahn J (2016). Topical delivery of 4-hexylresorcinol promotes wound healing via tumor necrosis factor-α suppression. Burns.

[17] Blaser H, Dostert C, Mak TW, Brenner D (2016). TNF and ROS Crosstalk in Inflammation. Trends Cell. Biol..

[18] Jo YY (2017). Accelerated biodegradation of silk sutures through matrix metalloproteinase activation by incorporating 4-hexylresorcinol. Sci. Rep..

[19] Kang YJ (2016). The effect of 4-hexylresorcinol on xenograft degradation in a rat calvarial defect model. Maxillofac. Plast. Reconstr. Surg..

[20] Lee SW, Um IC, Kim SG, Cha MS (2015). Evaluation of bone formation and membrane degradation in guided bone regeneration using a 4-hexylresorcinol-incorporated silk fabric membrane. Maxillofac. Plast. Reconstr. Surg..

[21] Kweon H, Kim SG, Choi JY (2014). Inhibition of foreign body giant cell formation by 4- hexylresorcinol through suppression of diacylglycerol kinase delta gene expression. Biomaterials.

[22] Lee SW (2013). Silk fibroin and 4-hexylresorcinol incorporation membrane for guided bone regeneration. J. Craniofac. Surg..

[23] Jo YY (2017). Bone regeneration is associated with the concentration of tumour necrosis factor-α induced by sericin released from a silk mat. Sci. Rep..

[24] Kundu B, Kurland NE, Yadavalli VK, Kundu SC (2014). Isolation and processing of silk proteins for biomedical applications. Int. J. Biol. Macromol..

[25] Haddad JJ, Harb HL (2005). Cytokines and the regulation of hypoxia-inducible factor (HIF)-1alpha. Int. Immunopharmacol..

[26] Shoeibi S, Mozdziak P, Mohammadi S (2018). Important signals regulating coronary artery angiogenesis. Microvasc. Res..

[27] Peyssonnaux C (2007). Cutting edge: essential role of hypoxia inducible factor-1α in development of lipopolysaccharide-induced sepsis. J. Immunol..

[28] Bergers G (2000). Matrix metalloproteinase-9 triggers the angiogenic switch during carcinogenesis. Nat. Cell. Biol..

[29] Deryugina EI, Soroceanu L, Strongin AY (2002). Up-regulation of vascular endothelial growth factor by membrane-type 1 matrix metalloproteinase stimulates human glioma xenograft growth and angiogenesis. Cancer Res..

[30] Liu XQ (2014). Specific Matrix metalloproteinases play different roles in intraplaque angiogenesis and plaque instability in rabbits. PLoS ONE.

[31] Eltzschig HK, Bratton DL, Colgan SP (2014). Targeting hypoxia signalling for the treatment of ischaemic and inflammatory diseases. Nat. Rev. Drug Discov..

[32] Barros MHM, Hauck F, Dreyer JH, Kempkes B, Niedobitek G (2013). Macrophage polarisation: an immunohistochemical approach for identifying M1 and M2 macrophages. PLoS ONE.

[33] Kamper P (2011). Tumor-infiltrating macrophages correlate with adverse prognosis and Epstein-Barr virus status in classical Hodgkin’s lymphoma. Haematologica.

[34] Zaki MA (2011). Prognostic implication of types of tumor-associated macrophages in Hodgkin lymphoma. Virchows Arch..

[35] Nawaz A (2017). CD206(+) M2-like macrophages regulate systemic glucose metabolism by inhibiting proliferation of adipocyte progenitors. Nat. Commun..

[36] Albina JE (2001). HIF-1 expression in healing wounds: HIF-1alpha induction in primary inflammatory cells by TNF-alpha. Am. J. Physiol. Cell. Physiol..

[37] Pouyssegur J, Mechta-Grigoriou F (2006). Redox regulation of the hypoxia-inducible factor. Biol. Chem..

[38] Brauchle M, Funk JO, Kind P, Werner S (1996). Ultraviolet B and H_2_O_2_ are potent inducers of vascular endothelial growth factor expression in cultured keratinocytes. J. Biol. Chem..

[39] Haddad JJ, Land SC (2001). A non-hypoxic, ROS sensitive pathway mediates TNF-α-dependent regulation of HIF-1α. FEBS Lett..

[40] Shah YM (2008). Hypoxia-inducible factor augments experimental colitis through an MIF-dependent inflammatory signaling cascade. Gastroenterology.

[41] Eltzschig HK, Carmeliet P (2011). Hypoxia and inflammation. N. Engl. J. Med..

[42] Okumura CY (2012). A new pharmacological agent (AKB-4924) stabilizes hypoxia inducible factor-1 (HIF-1) and increases skin innate defenses against bacterial infection. J. Mol. Med..

[43] Rajagopalan S (2007). Use of a constitutively active hypoxia-inducible factor-1α transgene as a therapeutic strategy in no-option critical limb ischemia patients: Phase I dose-escalation experience. Circulation.

[44] Kaelin WG (2007). Von Hippel–Lindau disease. An important review paper that summarizes the discovery and functional role of the VHL gene in the post-translational regulation of HIF levels. Annu. Rev. Pathol..

[45] Park JH (2003). Gastric epithelial reactive oxygen species prevent normoxic degradation of hypoxia-inducible factor-1alpha in gastric cancer cells. Clin. Cancer Res..

[46] Yen GC, Duh PD, Lin CW (2003). Effects of resveratrol and 4-hexylresorcinol on hydrogen peroxide-induced oxidative DNA damage in human lymphocytes. Free Radic. Res..

[47] Du HT, Du LL, Tang XL, Ge HY, Liu P (2017). Blockade of MMP-2 and MMP-9 inhibits corneal lymphangiogenesis. Graefes Arch. Clin. Exp. Ophthalmol..

[48] Du HT, Liu P (2016). Matrix metalloproteinase 14 participates in corneal lymphangiogenesis through the VEGF-C/VEGFR-3 signaling pathway. Exp. Ther. Med..

[49] Bruno A (2018). Angiogenin and the MMP9-TIMP2 axis are up-regulated in proangiogenic, decidual NK-like cells from patients with colorectal cancer. FASEB J..

[50] Wang M (2012). Chronic matrix metalloproteinase inhibition retards age-associated arterial proinflammation and increase in blood pressure. Hypertension.

[51] Woenne EC (2010). MMP inhibition blocks fibroblast-dependent skin cancer invasion, reduces vascularization and alters VEGF-A and PDGF-BB expression. Anticancer Res..

[52] Xie Y (2017). Nuclear matrix metalloproteinases: functions resemble the evolution from the intracellular to the extracellular compartment. Cell Death Discov..

[53] Mannello F, Medda V (2012). Nuclear localization of matrix metalloproteinases. Prog. Histochem. Cytochem..

[54] Okamoto T (1997). Activation of human neutrophil procollagenase by nitrogen dioxide and peroxynitrite: a novel mechanism for procollagenase activation involving nitric oxide. Arch. Biochem. Biophys..

[55] Sounni NE (2004). Up-regulation of vascular endothelial growth factor-A by active membrane-type 1 matrix metalloproteinase through activation of Src-tyrosine kinases. J. Biol. Chem..

[56] Ware JA, Simons M (1997). Angiogenesis in ischemic heart disease. Nat. Med..

[57] Sun Y (2003). VEGF-induced neuroprotection, neurogenesis, and angiogenesis after focal cerebral ischemia. J. Clin. Invest..

[58] Zechariah A (2013). Vascular endothelial growth factor promotes pericyte coverage of brain capillaries, improves cerebral blood flow during subsequent focal cerebral ischemia, and preserves the metabolic penumbra. Stroke.

[59] Kim CW (2018). 4-Hexylresorcinol-incorporated silk vascular patch in rat carotid defect model. Appl. Sci..

[60] Ramakrishnan S, Anand V, Roy S (2014). Vascular endothelial growth factor signaling in hypoxia and inflammation. J. Neuroimmune Pharmacol..

[61] Wang XH, Cavell BE, Syed Alwi SS, Packham G (2009). Inhibition of hypoxia inducible factor by phenethyl isothiocyanate. Biochem. Pharmacol..

[62] Deiana M (2017). Derangement of intestinal epithelial cell monolayer by dietary cholesterol oxidation products. Free Radic. Biol. Med..

