# Supplementary material for: 4-Hexylresorcinol and silk sericin increase the expression of vascular endothelial growth factor via different pathways
Source: Sci Rep. 2019 Mar 5;9:3448. doi: 10.1038/s41598-019-40027-5 (PMC6400942; doi:10.1038/s41598-019-40027-5)
Supplement: Supplementary file 1 — Supplementary information [file 41598_2019_40027_MOESM1_ESM.docx]

**4-Hexylresorcinol and silk sericin increase the expression of vascular endothelial growth factor via different pathways**

You-Young Jo^1, #^, Dae-Won Kim^2, #^, Je-Yong Choi^3^, and Seong-Gon Kim^4, *^

^1^Sericultural and Apicultural Division, National Institute of Agricultural Science, RDA, Wanju 55365, Republic of Korea

^2^Dept. of Oral Biochemistry, College of Dentistry, Gangneung-Wonju National University, Gangneung 28644, Republic of Korea

^3^School of Biochemistry and Cell Biology, BK21 Plus KNU Biomedical Convergence Program, Skeletal Diseases Analysis Center, Korea Mouse Phenotyping Center (KMPC), Kyungpook National University, Daegu 41944, Korea

^4^Dept. of Oral and Maxillofacial Surgery, College of Dentistry, Gangneung-Wonju National University, Gangneung 28644, Republic of Korea

^#^Both authors contributed equally.

*Corresponding author

E-mail address: kimsg@gwnu.ac.kr, Fax: +82-33-641-2477

**Supplementary Figure 1. The action of MMP-2 inhibitor (ARP100) for 4HR induced angiogenesis.** (A) The expression level of MMP-2 was decreased by the application of ARP100. However, its expression level was recovered at 40 nM of ARP100 application. The concentration of IC50 for MMP-2 was 12 nM. As ARP100 inhibits the catalytic activity of MMP-2, the change of MMP-2 expression level was interesting. (B) 30 nM of ARP100 could not inhibit 4HR induced VEGF-A expression. As 30 nM of ARP100 could inhibit both the catalytic activity of MMP-2 and MMP-2 expression, MMP-2 did not seem to be involved in 4HR induced angiogenesis.

**A. Determination of optimal ARP100 concentration for inhibiting MMP-2**


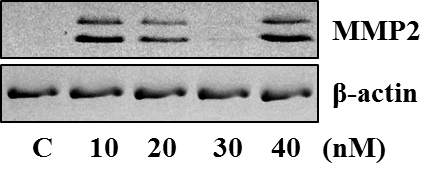


**B. The action of ARP100 for 4HR induced angiogenesis.**

**
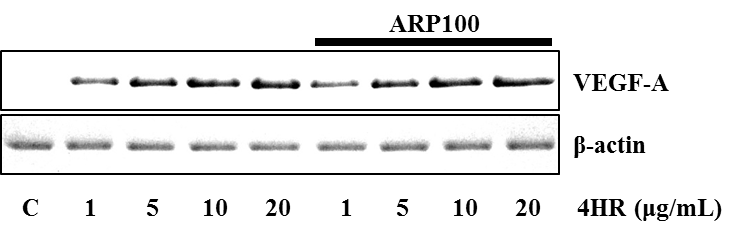
**

**Supplementary Figure 2. Increased expression of MMP-13 and MMP-14 by the administration of 4HR.** (A) The expression level of MMP-13 was increased by the application of 4HR. (B) The expression level of MMP-14 was increased by the application of 4HR.

**A. The expression of MMP-13 was increased by 4HR treatment.**

**
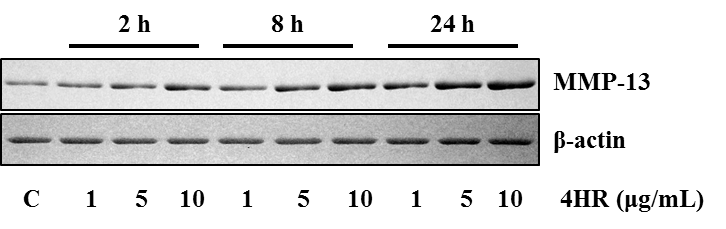
**

**B. The expression of MMP-14 was increased by 4HR treatment.**

**
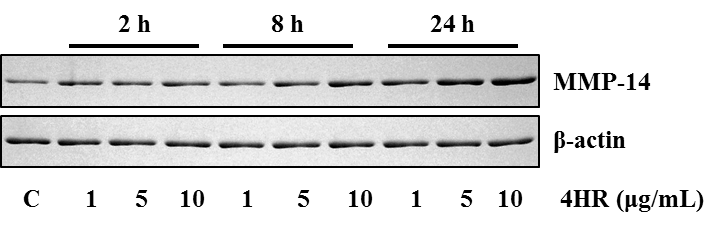
**

**Supplementary Figure 3. Determination of PEITC concentration and expression of vascular endothelial growth factor A (VEGF-C)/ angiogenin with or without PEITC.** (A) 10 μM of PEITC could inhibit H_2_O_2_ induced HIF-1α expression.

**
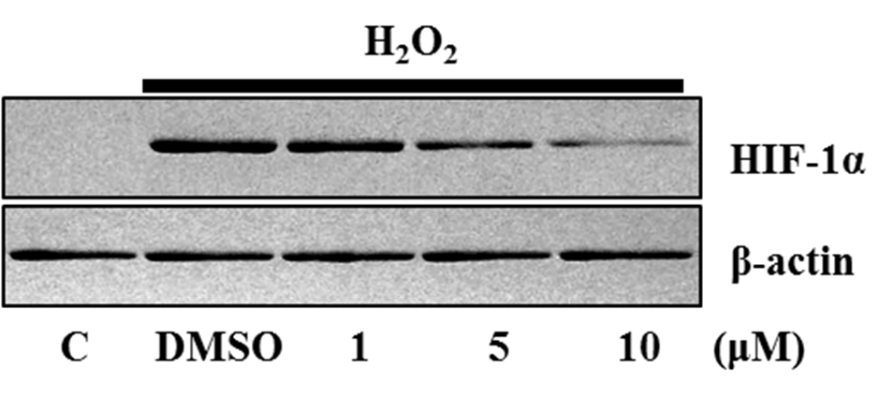
**

(B) Pretreatment with PEITC decreased the sericin-induced expression of VEGF-C/angiogenin. However, PEITC did not inhibit the 4-hexylresorcinol (4HR)-induced expression of VEGF-C/ angiogenin.


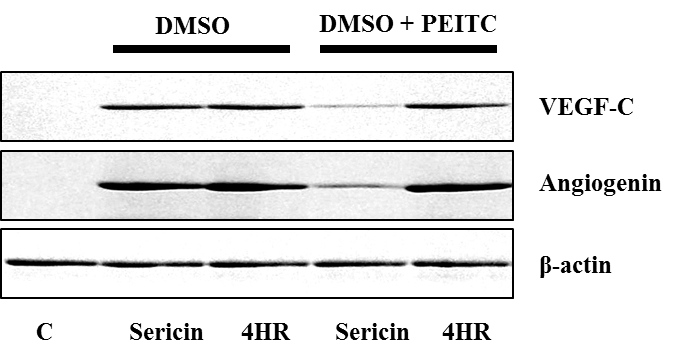


**Supplementary Figure 4. Determination of optimal PD166793 concentration for inhibiting MMPs and expression of vascular endothelial growth factor A (VEGF-C)/ angiogenin with or without PD166793.** (A) The expression level of MMP-13 was decreased by the application of PD166793. PD166793 has been known to inhibit the catalytic activity of MMP-1, MMP-2, MMP-3, MMP-7, MMP-9, MMP-13, and MMP-14. IC50s are 6.1, 0.047, 0.012, 7.2, 7.9, 0.008, and 0.24 μM for MMP-1, MMP-2, MMP-3, MMP-7, MMP-9, MMP-13, and MMP-14, respectively. The expression level of MMP-13 was recovered at 25 nM of PD166793 application. As the concentration of IC50 for MMP-3 and MMP-13 was 12 nM and 8 nM, respectively, 20 nM of PD166793 can inhibit the catalytic activity of MMP-3 and MMP-13. In addition, it can inhibit MMP-13 expression.


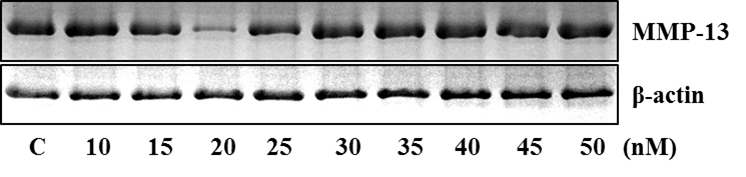


(B) Expression of vascular endothelial growth factor C (VEGF-C)/ angiogenin with or without pretreatment with the matrix metalloproteinase (MMP) inhibitor PD166793. Pretreatment with PD166793 decreased the 4-hexylresorcinol (4HR)-induced expression of VEGF-C/ angiogenin.


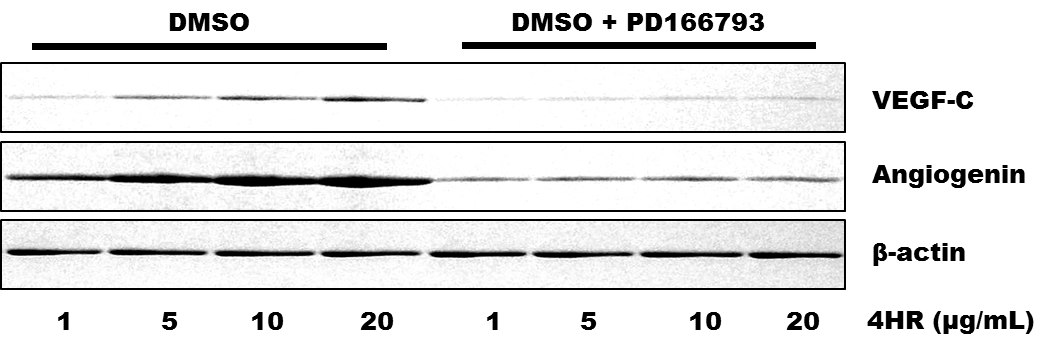


**Supplementary Figure 5. Immunofluorescence image for von Willebrand factor (vWF).** The expressions of vWF were mainly observed in the blood cells. Interestingly, the expressions of vWF were frequently observed in the endothelium in silk mat incorporated with 4HR (arrows). Silk sericin (*) was also shown fluorescence (original magnification x 200, DAPI: 4, 6-diamidino-2-phenylindole).

**
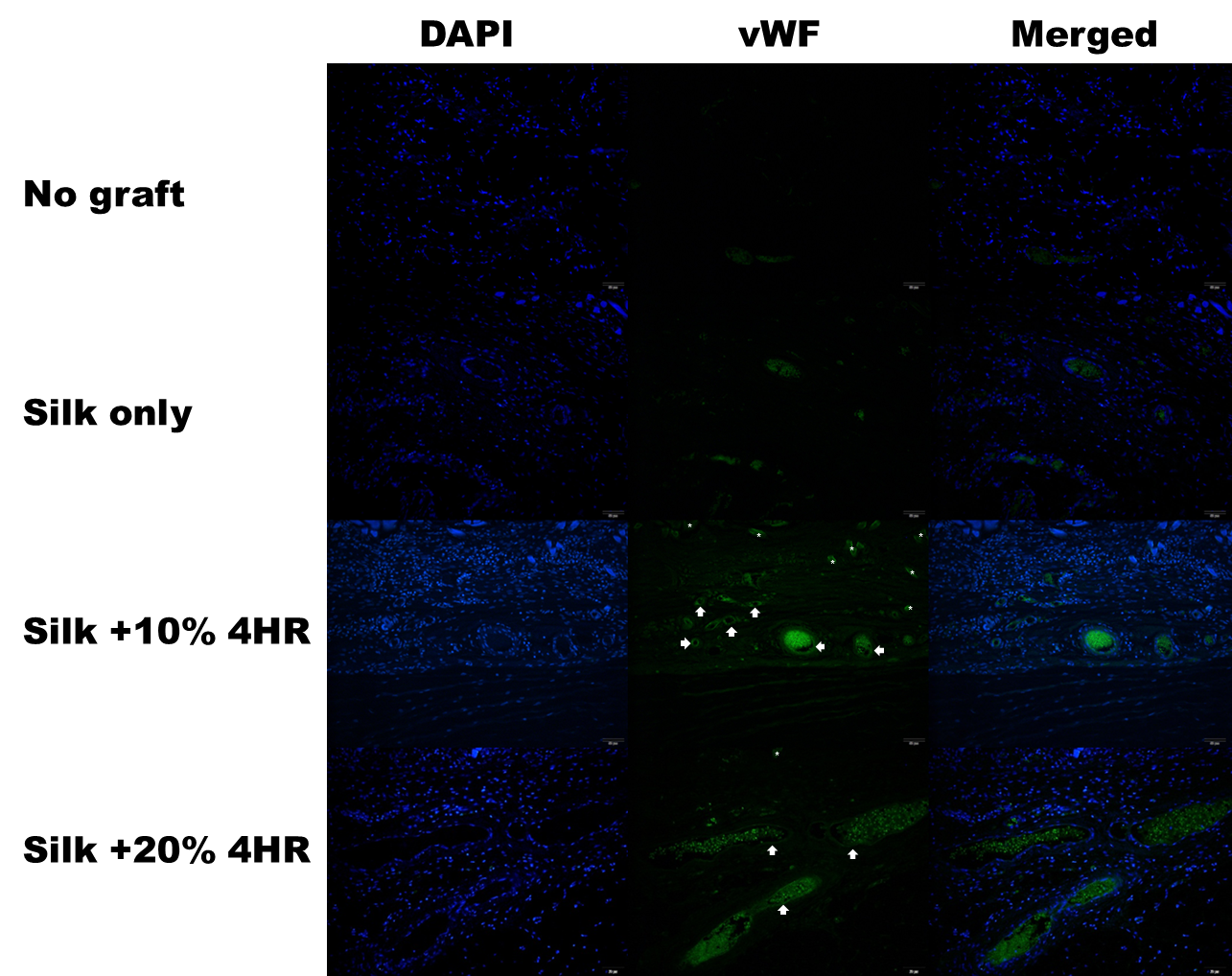
**

**Supplementary Figure 6. Full length blot of Figure 1A**

**Sericin induced angiogenesis**

A. β-actin (from left lane to right, **1:** no-treatment, **2:** 1 μg/mL sericin at 2 h, **3:** 5 μg/mL sericin at 2 h, **4:** 10 μg/mL sericin at 2 h, **5:** 1 μg/mL sericin at 8 h, **6:** 5 μg/mL sericin at 8 h, **7:** 10 μg/mL sericin at 8 h, **8:** 1 μg/mL sericin at 24 h, **9:** 5 μg/mL sericin at 24 h, **10:** 10 μg/mL sericin at 24 h)

**
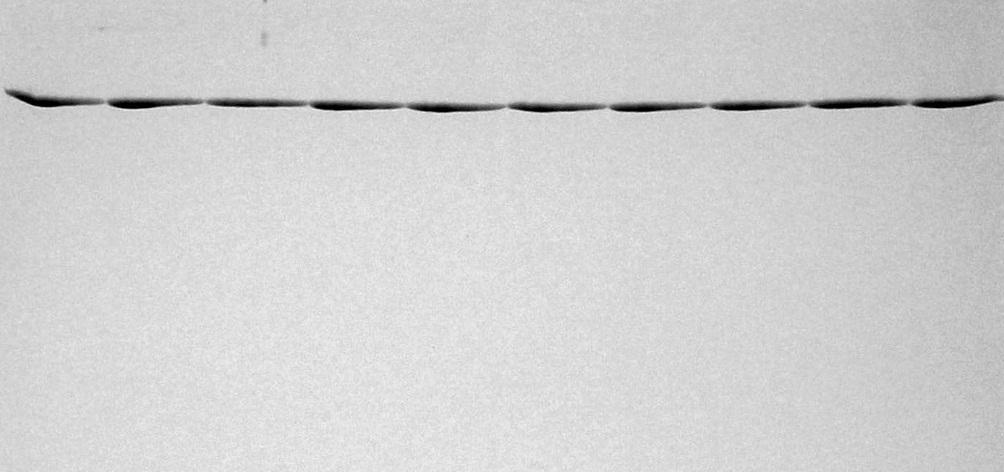
**

B. Vascular endothelial growth factor-A (from left lane to right, **1:** no-treatment, **2:** 1 μg/mL sericin at 2 h, **3:** 5 μg/mL sericin at 2 h, **4:** 10 μg/mL sericin at 2 h, **5:** 1 μg/mL sericin at 8 h, **6:** 5 μg/mL sericin at 8 h, **7:** 10 μg/mL sericin at 8 h, **8:** 1 μg/mL sericin at 24 h, **9:** 5 μg/mL sericin at 24 h, **10:** 10 μg/mL sericin at 24 h)


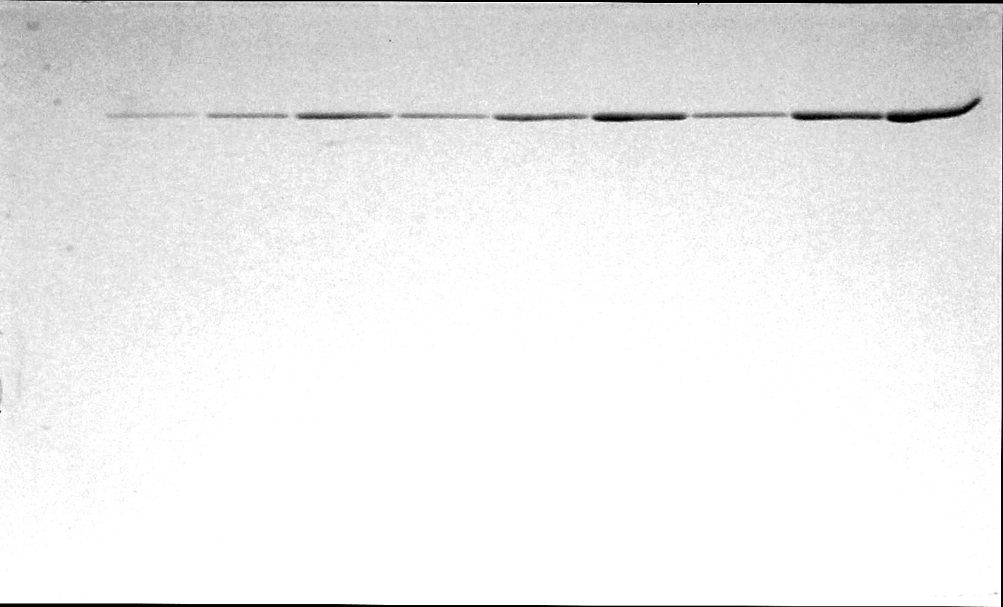


C. Vascular endothelial growth factor-C (from left lane to right, **1:** no-treatment, **2:** 1 μg/mL sericin at 2 h, **3:** 5 μg/mL sericin at 2 h, **4:** 10 μg/mL sericin at 2 h, **5:** 1 μg/mL sericin at 8 h, **6:** 5 μg/mL sericin at 8 h, **7:** 10 μg/mL sericin at 8 h, **8:** 1 μg/mL sericin at 24 h, **9:** 5 μg/mL sericin at 24 h, **10:** 10 μg/mL sericin at 24 h)


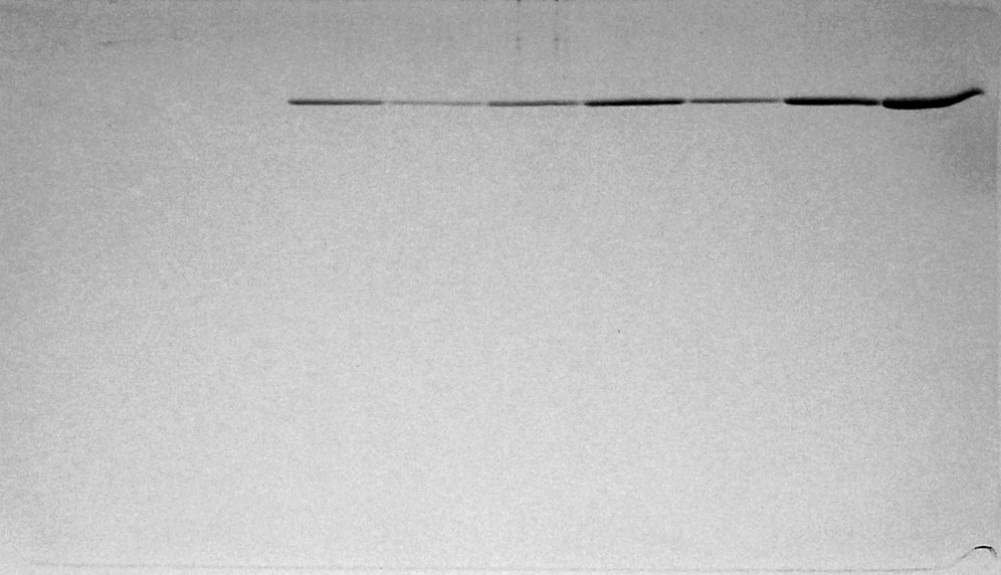


D. Angiogenin (from left lane to right, **1:** no-treatment, **2:** 1 μg/mL sericin at 2 h, **3:** 5 μg/mL sericin at 2 h, **4:** 10 μg/mL sericin at 2 h, **5:** 1 μg/mL sericin at 8 h, **6:** 5 μg/mL sericin at 8 h, **7:** 10 μg/mL sericin at 8 h, **8:** 1 μg/mL sericin at 24 h, **9:** 5 μg/mL sericin at 24 h, **10:** 10 μg/mL sericin at 24 h)


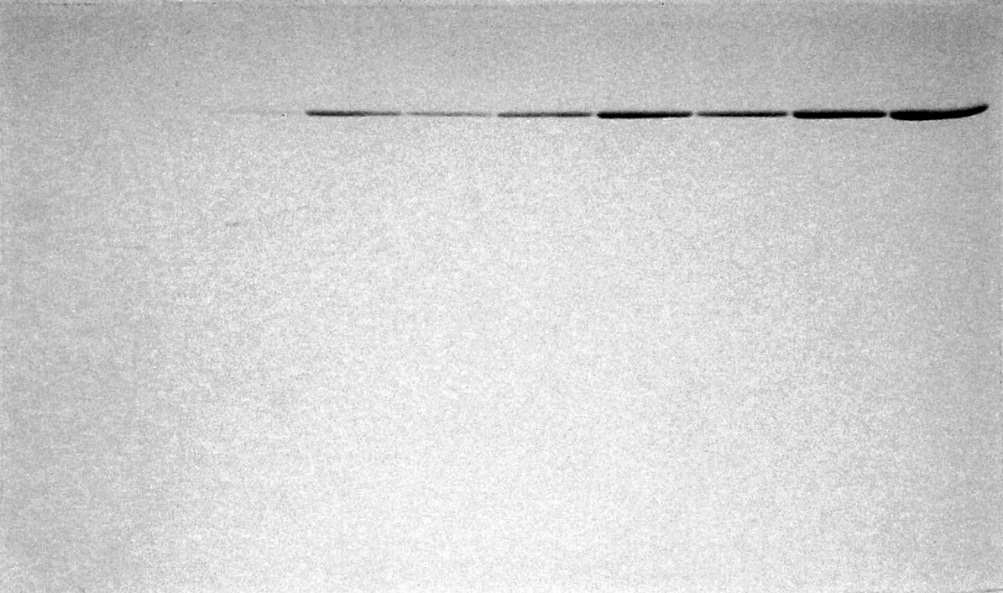


**Supplementary Figure 7. Full length blot of Figure 1B**

**4-Hexylresorcinol (4HR) induced angiogenesis**

A. β-actin (from left lane to right, **1:** no-treatment, **2:** 1 μg/mL 4HR at 2 h, **3:** 5 μg/mL 4HR at 2 h, **4:** 10 μg/mL 4HR at 2 h, **5:** 1 μg/mL 4HR at 8 h, **6:** 5 μg/mL 4HR at 8 h, **7:** 10 μg/mL 4HR at 8 h, **8:** 1 μg/mL 4HR at 24 h, **9:** 5 μg/mL 4HR at 24 h, **10:** 10 μg/mL 4HR at 24 h)


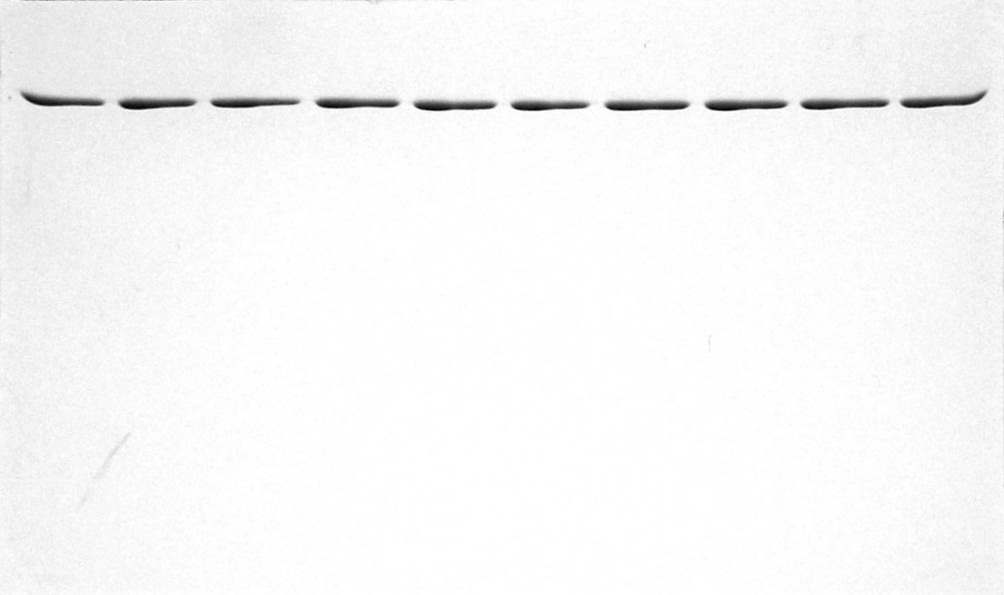


B. Vascular endothelial growth factor-A (from left lane to right, **1:** no-treatment, **2:** 1 μg/mL 4HR at 2 h, **3:** 5 μg/mL 4HR at 2 h, **4:** 10 μg/mL 4HR at 2 h, **5:** 1 μg/mL 4HR at 8 h, **6:** 5 μg/mL 4HR at 8 h, **7:** 10 μg/mL 4HR at 8 h, **8:** 1 μg/mL 4HR at 24 h, **9:** 5 μg/mL 4HR at 24 h, **10:** 10 μg/mL 4HR at 24 h)


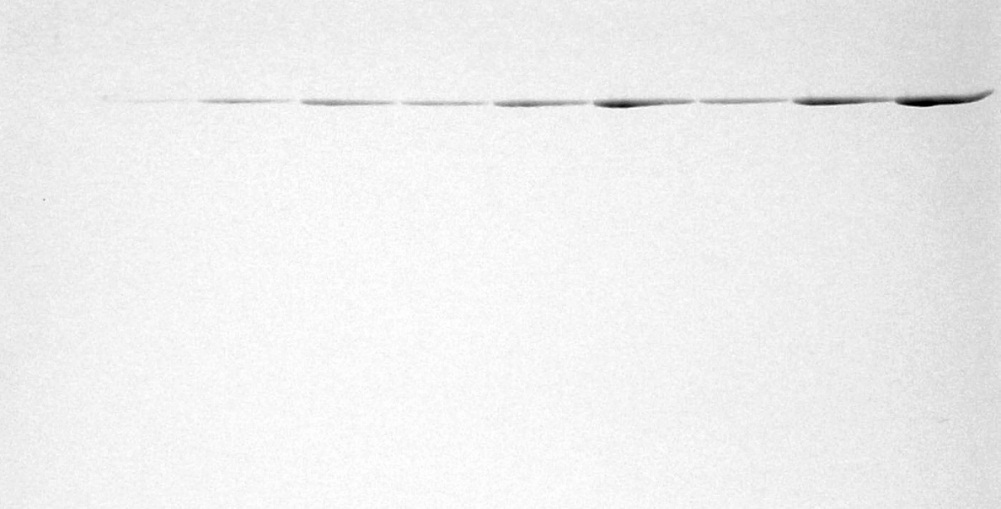


C. Vascular endothelial growth factor-C (from left lane to right, **1:** no-treatment, **2:** 1 μg/mL 4HR at 2 h, **3:** 5 μg/mL 4HR at 2 h, **4:** 10 μg/mL 4HR at 2 h, **5:** 1 μg/mL 4HR at 8 h, **6:** 5 μg/mL 4HR at 8 h, **7:** 10 μg/mL 4HR at 8 h, **8:** 1 μg/mL 4HR at 24 h, **9:** 5 μg/mL 4HR at 24 h, **10:** 10 μg/mL 4HR at 24 h)


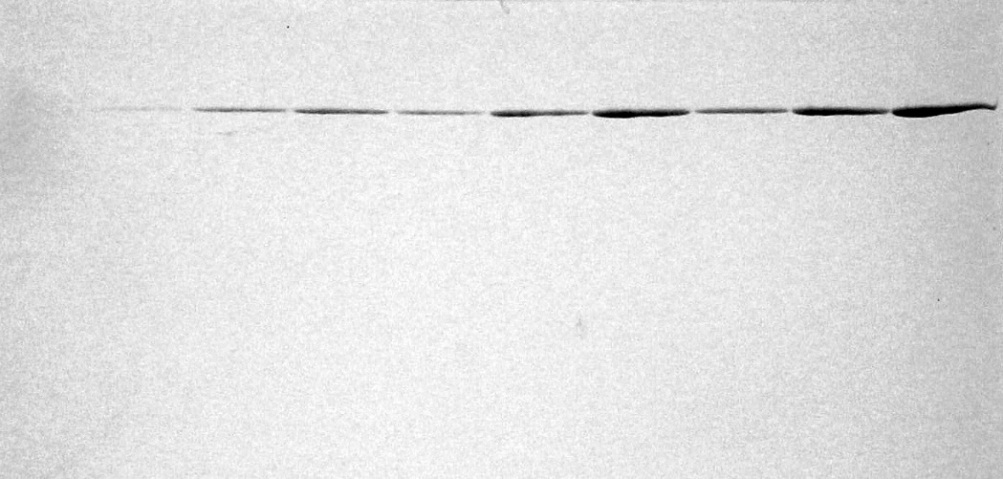


D. Angiogenin (from left lane to right, **1:** no-treatment, **2:** 1 μg/mL 4HR at 2 h, **3:** 5 μg/mL 4HR at 2 h, **4:** 10 μg/mL 4HR at 2 h, **5:** 1 μg/mL 4HR at 8 h, **6:** 5 μg/mL 4HR at 8 h, **7:** 10 μg/mL 4HR at 8 h, **8:** 1 μg/mL 4HR at 24 h, **9:** 5 μg/mL 4HR at 24 h, **10:** 10 μg/mL 4HR at 24 h)


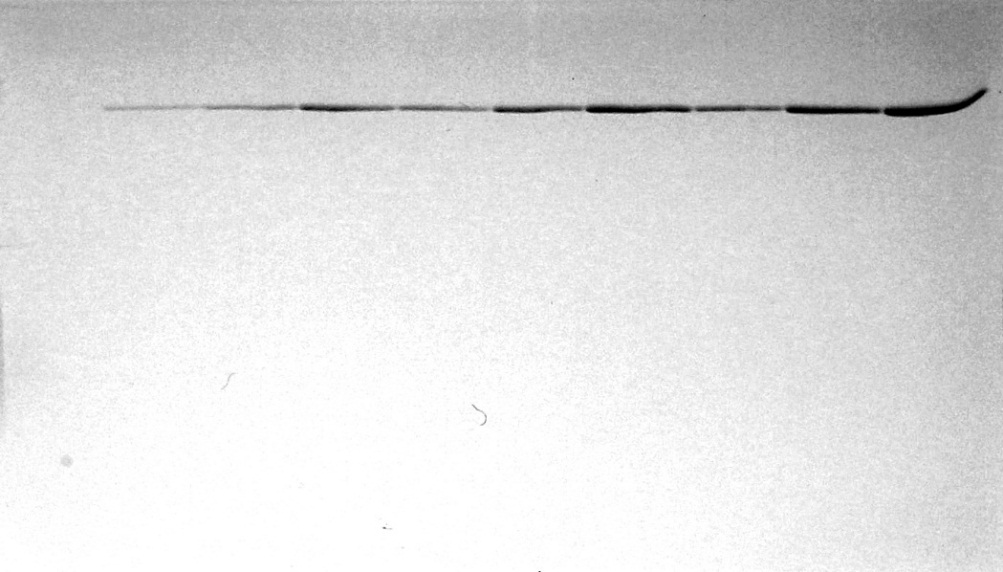


**Supplementary Figure 8. Full length blot of Figure 2A**

**Hypoxia induced factor-1α (HIF-1α)**

A. β-actin (from left lane to right, **1:** no-treatment, **2:** 10 μg/mL sericin at 2 h, **3:** 10 μg/mL sericin at 8 h, **4:** 10 μg/mL sericin at 24 h, **5:** no-treatment, **6:** 10 μg/mL 4-hexylresorcinol (4HR) at 2 h, **7:** 10 μg/mL 4HR at 8 h, **8:** 10 μg/mL 4HR at 24 h)

**
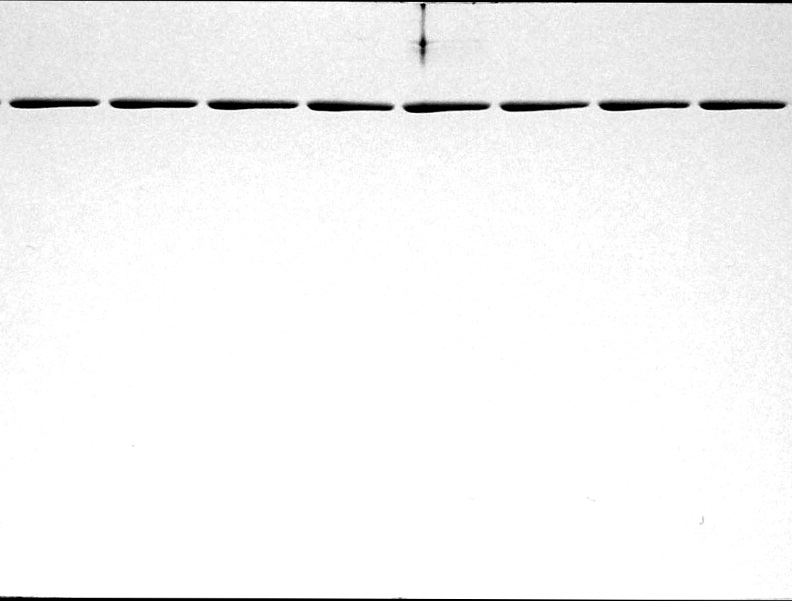
**

B. HIF-1α (from left lane to right, **1:** no-treatment, **2:** 10 μg/mL sericin at 2 h, **3:** 10 μg/mL sericin at 8 h, **4:** 10 μg/mL sericin at 24 h, **5:** no-treatment, **6:** 10 μg/mL 4-hexylresorcinol (4HR) at 2 h, **7:** 10 μg/mL 4HR at 8 h, **8:** 10 μg/mL 4HR at 24 h)


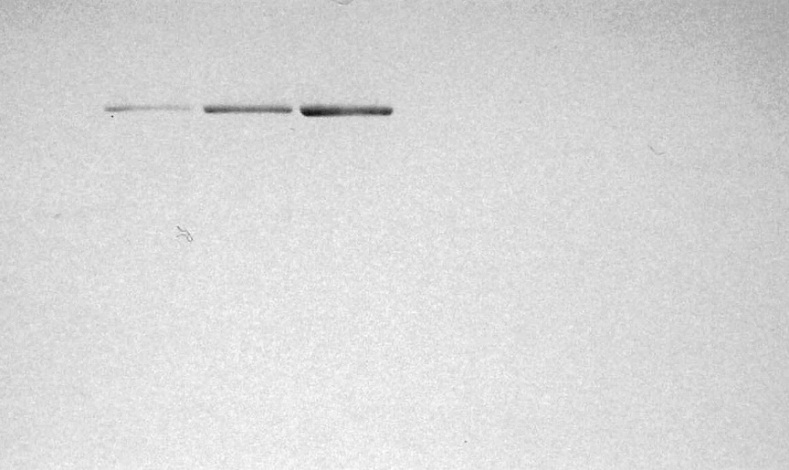


**Supplementary Figure 9. Full length blot of Figure 2B**

**Hypoxia induced factor-2α**

A. β-actin (from left lane to right, **1:** no-treatment, **2:** 10 μg/mL sericin at 2 h, **3:** 10 μg/mL sericin at 8 h, **4:** 10 μg/mL sericin at 24 h, **5:** no-treatment, **6:** 10 μg/mL 4-hexylresorcinol (4HR) at 2 h, **7:** 10 μg/mL 4HR at 8 h, **8:** 10 μg/mL 4HR at 24 h)

**
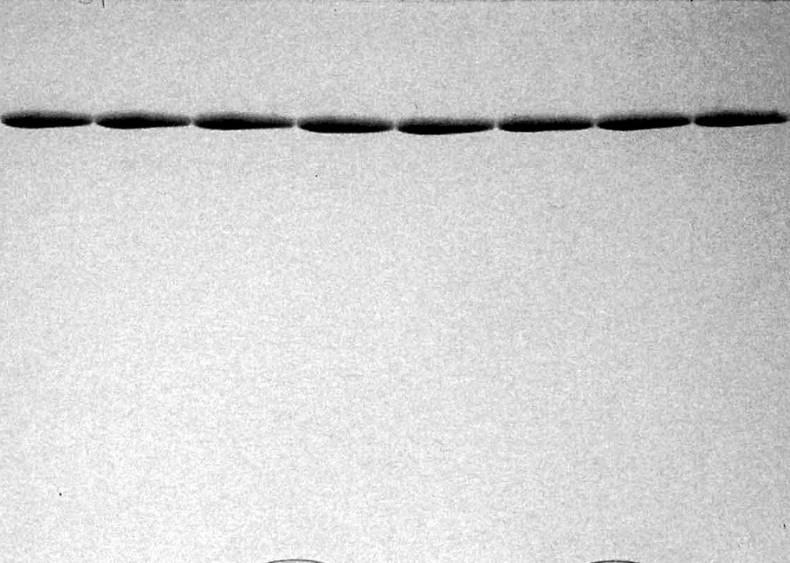
**

B. HIF-2α (from left lane to right, **1:** no-treatment, **2:** 10 μg/mL sericin at 2 h, **3:** 10 μg/mL sericin at 8 h, **4:** 10 μg/mL sericin at 24 h, **5:** no-treatment, **6:** 10 μg/mL 4-hexylresorcinol (4HR) at 2 h, **7:** 10 μg/mL 4HR at 8 h, **8:** 10 μg/mL 4HR at 24 h)

**
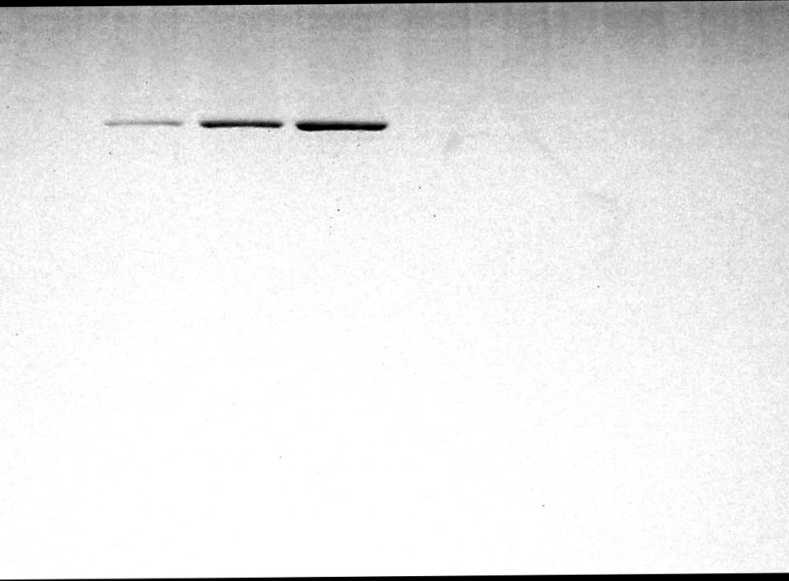
**

**Supplementary Figure 10. Full length blot of Figure 3**

**The effects of PEITC on VEGF expression. PEITC could inhibit sericin induced VEGF expression, but could not 4-hexylresorcinol induced VEGF expression.**

A. β-actin (from left lane to right, 1: no-treatment, 2: 10 μg/mL sericin, 3: 10 μg/mL 4-hexylresorcinol (4HR), 4: 10 μg/mL sericin with PEITC, 5: 10 μg/mL 4HR with PEITC)

**
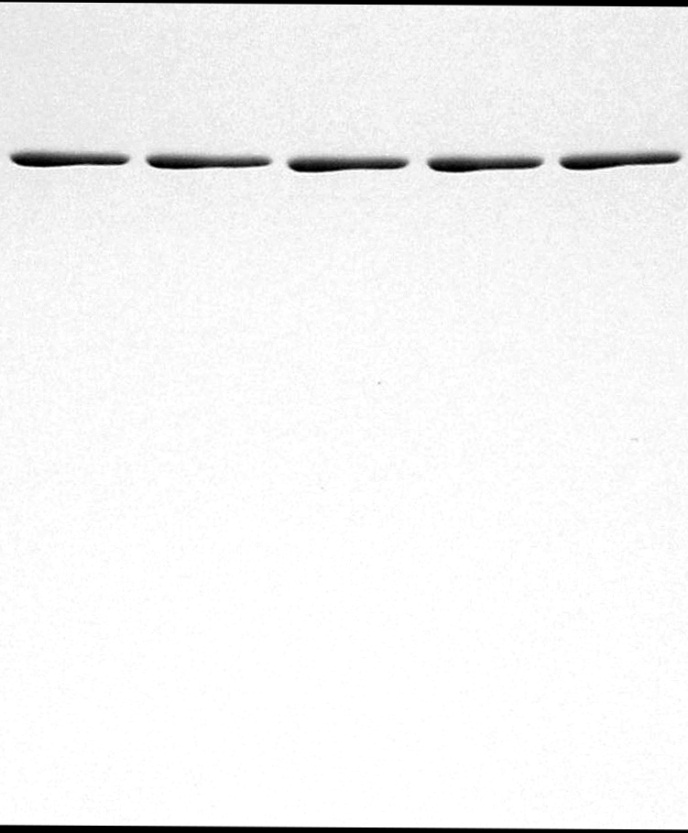
**

B. Vascular endothelial growth factor (from left lane to right, 1: no-treatment, 2: 10 μg/mL sericin, 3: 10 μg/mL 4-hexylresorcinol (4HR), 4: 10 μg/mL sericin with PEITC, 5: 10 μg/mL 4HR with PEITC)

**
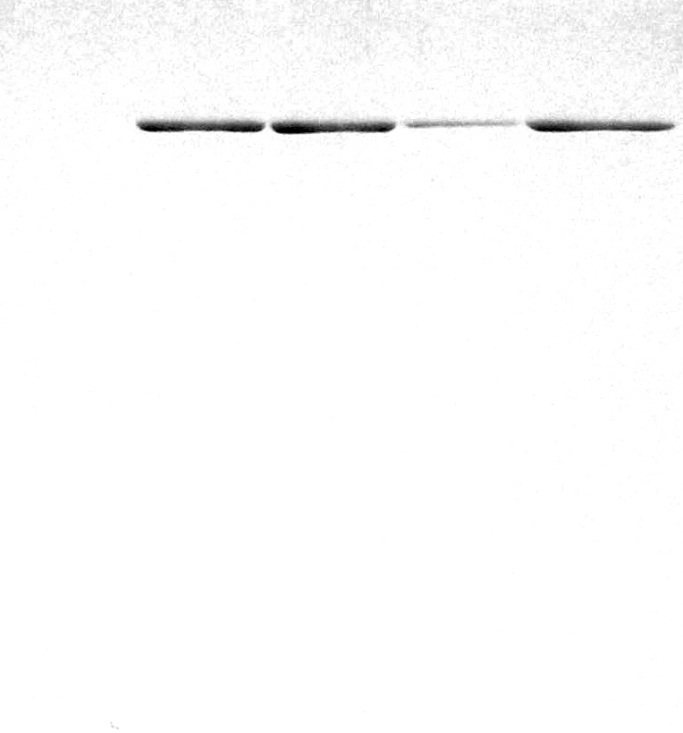
**

**Supplementary Figure 11. Full length blot of Figure 4A**

**The action of MMP inhibitor (PD166703) for 4HR induced angiogenesis**

A. β-actin (from left lane to right, 1: no-treatment, 2: 1 μg/mL 4-hexylresorcinol (4HR), 3: 5 μg/mL 4HR, 4: 10 μg/mL 4HR, 5: 20 μg/mL 4HR, 6: 1 μg/mL 4HR with PD166793 pre-treatment, 7: 5 μg/mL 4HR with PD166793 pre-treatment, 8: 10 μg/mL 4HR with PD166793 pre-treatment, and 8: 20 μg/mL 4HR with PD166793 pre-treatment)


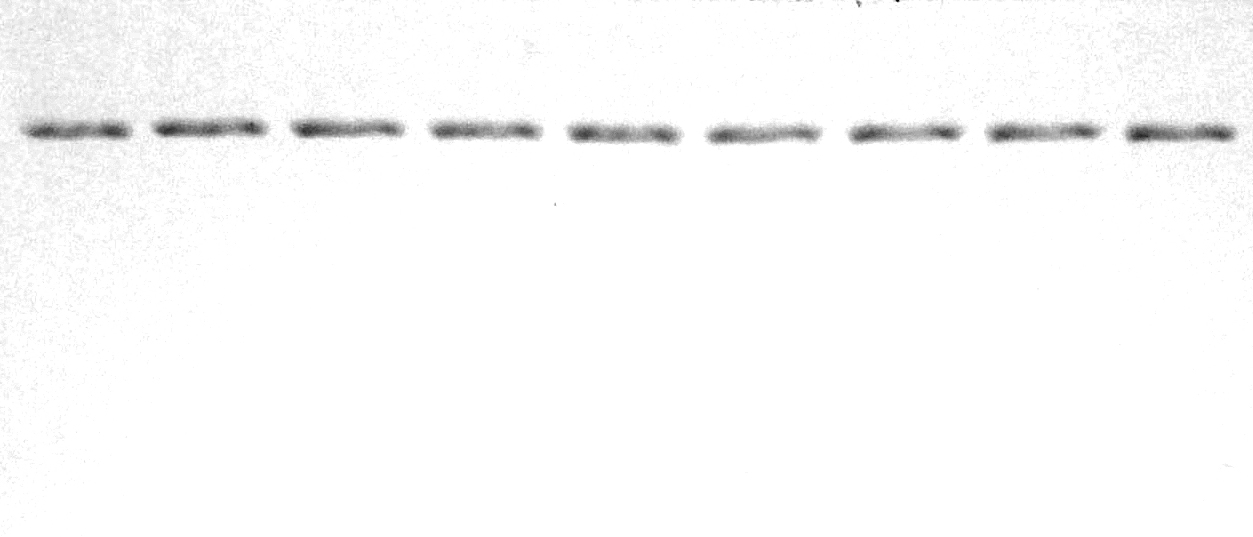


B. Vascular endothelial growth factor-A (from left lane to right, 1: no-treatment, 2: 1 μg/mL 4-hexylresorcinol (4HR), 3: 5 μg/mL 4HR, 4: 10 μg/mL 4HR, 5: 20 μg/mL 4HR, 6: 1 μg/mL 4HR with PD166793 pre-treatment, 7: 5 μg/mL 4HR with PD166793 pre-treatment, 8: 10 μg/mL 4HR with PD166793 pre-treatment, and 8: 20 μg/mL 4HR with PD166793 pre-treatment)


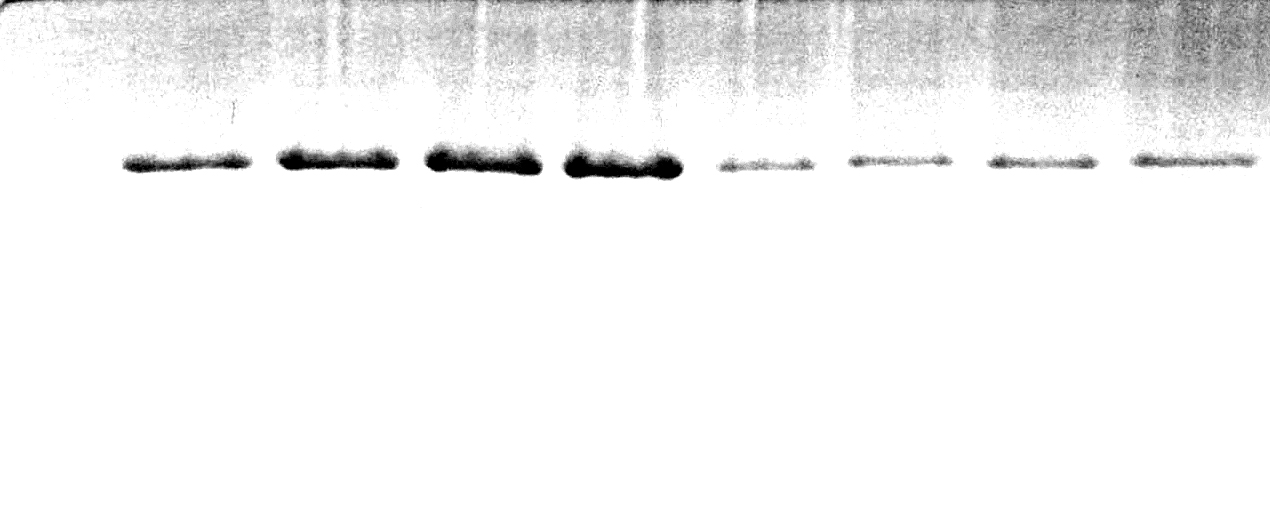


**Supplementary Figure 12. Full length blot of Figure 4B**

A. β-actin (from left lane to right, 1: no-pre-treatment, 2: 10 nM PD166793 pre-treatment and 10 μg/mL 4-hexylresorcinol (4HR), 3: 20 nM PD166793 pre-treatment and 10 μg/mL 4HR, 4: 50 nM PD166793 pre-treatment and 10 μg/mL 4HR 5: 100 nM PD166793 pre-treatment and 10 μg/mL 4HR 0 μg/mL 4HR)


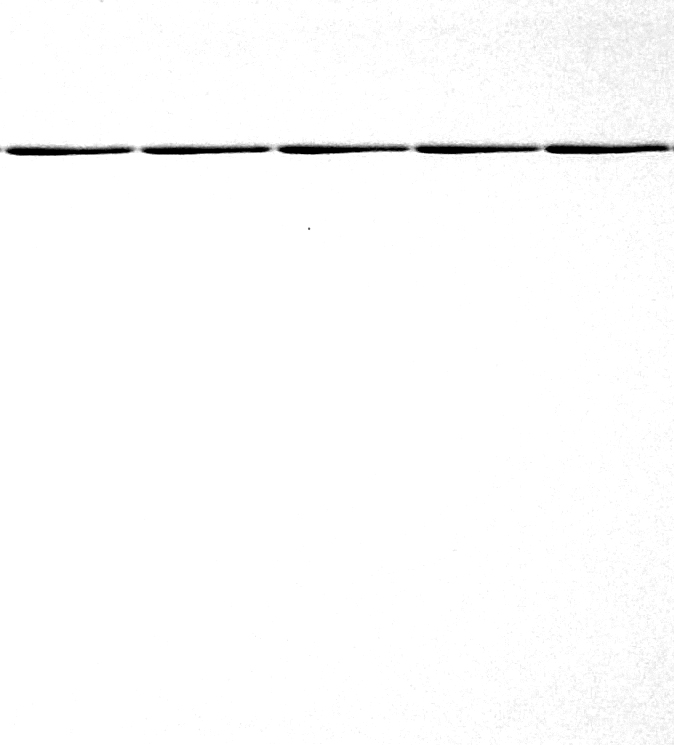


B. Vascular endothelial growth factor-A (from left lane to right, 1: no-pre-treatment, 2: 10 nM PD166793 pre-treatment and 10 μg/mL 4-hexylresorcinol (4HR), 3: 20 nM PD166793 pre-treatment and 10 μg/mL 4HR, 4: 50 nM PD166793 pre-treatment and 10 μg/mL 4HR 5: 100 nM PD166793 pre-treatment and 10 μg/mL 4HR 0 μg/mL 4HR)


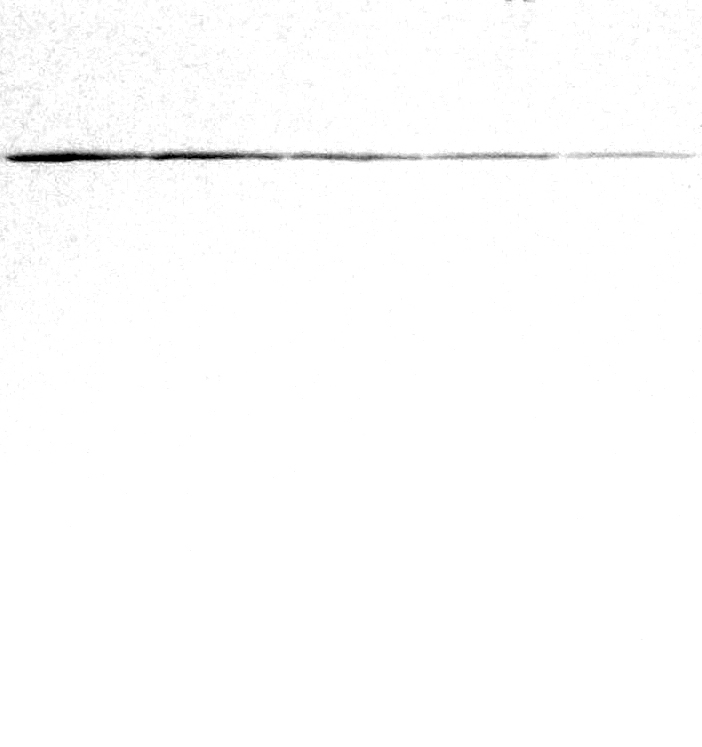


**Supplementary Figure 13. Full length blot of Figure 6**

The expression of M1/ M2 marker at 24 h after silk sericin (S) or 4-hexylresorcinol (4HR) administration.

A. β-actin (from left lane to right, 1: no-treatment, 2: 10 μg/mL S, 3: 10 μg/mL 4HR)


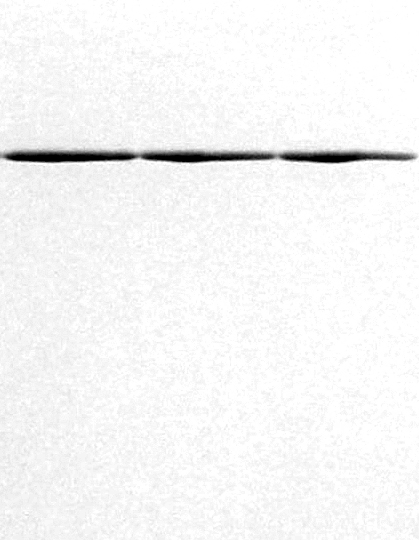


B. CD68 (from left lane to right, 1: no-treatment, 2: 10 μg/mL S, 3: 10 μg/mL 4HR)


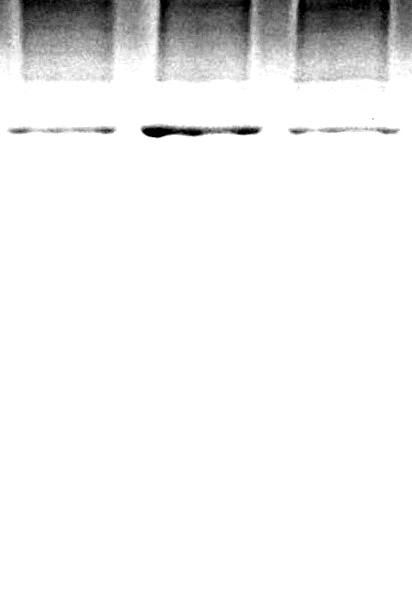


C. pStat1 (from left lane to right, 1: no-treatment, 2: 10 μg/mL S, 3: 10 μg/mL 4HR)

**
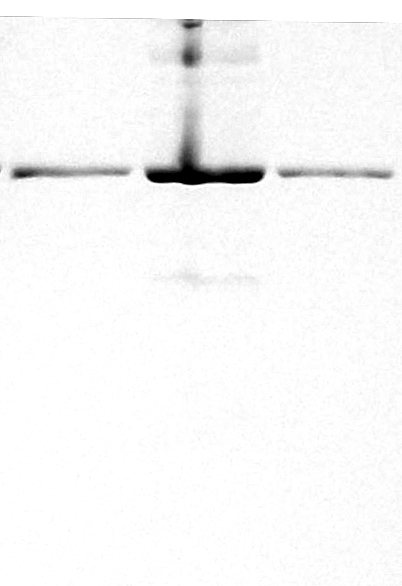
**

D. CD206 (from left lane to right, 1: no-treatment, 2: 10 μg/mL S, 3: 10 μg/mL 4HR)

**
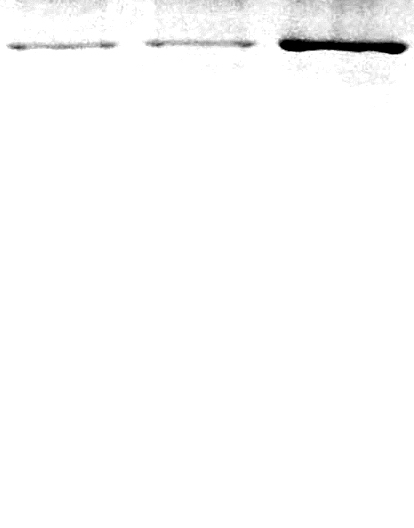
**

E. c-Maf (from left lane to right, 1: no-treatment, 2: 10 μg/mL S, 3: 10 μg/mL 4HR)

**
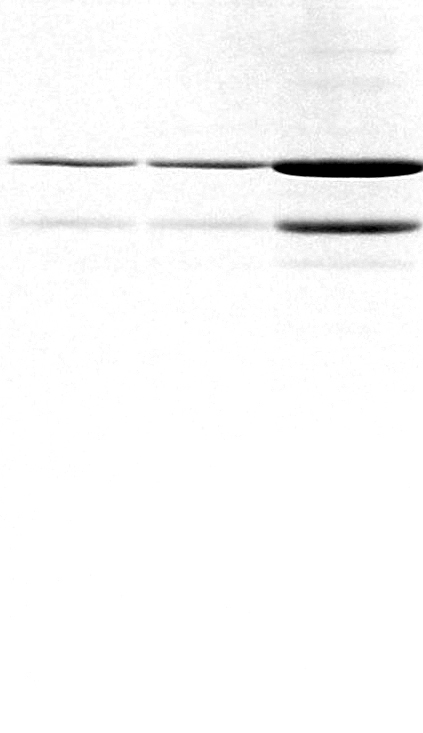
**

**Supplementary Figure 14. Full length blot of Figure 7C**

A. β-actin (from left lane to right, 1: no-treatment, 2: silk mat only, 3: 10 % 4-hexylresorcinol (4HR) + silk mat, and 4: 20 % 4HR + silk mat)


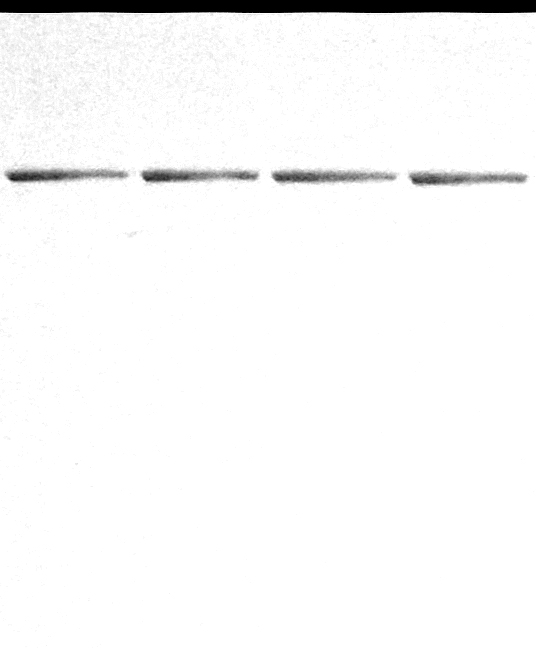


B. Vascular endothelial growth factor-A (VEGF-A) (1: no-treatment, 2: silk mat only, 3: 10 % 4HR + silk mat, and 4: 20 % 4HR + silk mat)


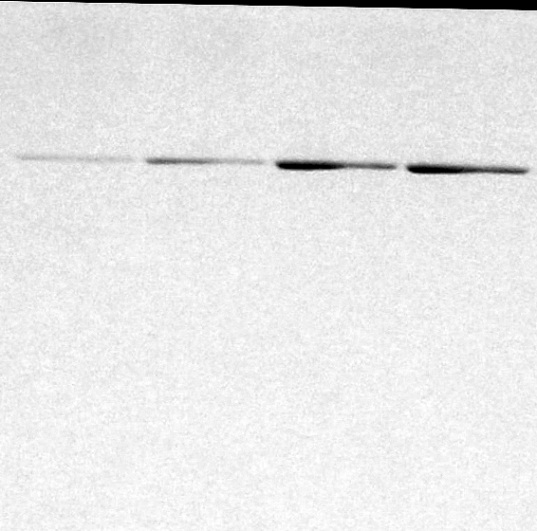


C. VEGF-C (from left lane to right, 1: no-treatment, 2: silk mat only, 3: 10 % 4HR + silk mat, and 4: 20 % 4HR + silk mat)


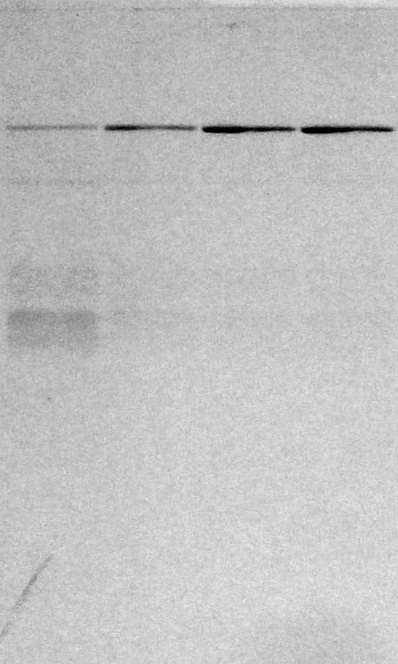


D. Angiogenin (from left lane to right, no-treatment, 1: no-treatment, 2: silk mat only, 3: 10 % 4HR+ silk mat, and 4: 20 % 4HR + silk mat)


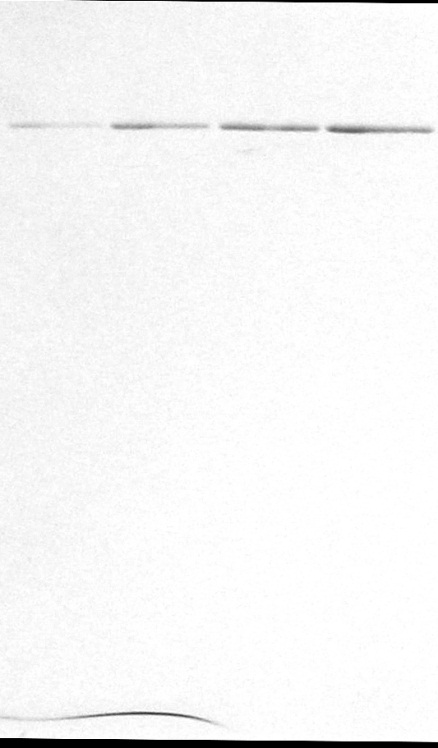


**Supplementary list for antibodies**

| Name | Vendor | Catalogue number | Host species |
| --- | --- | --- | --- |
| VEGF-A | Santa Cruz Biotechnology | sc-57496 | Mouse |
| VEGF-C | Santa Cruz Biotechnology | sc-374628 | Mouse |
| angiogenin | Santa Cruz Biotechnology | sc-74528 | Mouse |
| MMP-13 | Santa Cruz Biotechnology | sc-515284 | Mouse |
| MMP-14 | Santa Cruz Biotechnology | sc-373908 | Mouse |
| CD68 | Santa Cruz Biotechnology | sc-20060 | Mouse |
| CD206 | Santa Cruz Biotechnology | sc-70585 | Mouse |
| c-Maf | Santa Cruz Biotechnology | sc-293420 | Mouse |
| pSTAT1 | Santa Cruz Biotechnology | sc-8394 | Mouse |
| β-actin | Sigma-Aldrich | A5441 | Mouse |
| HIF-1α | Santa Cruz Biotechnology | sc-13515 | Mouse |
| HIF-2α | Santa Cruz Biotechnology | sc-13596 | Mouse |
| vWF | Santa Cruz Biotechnology | sc-365712 | Mouse |

(VEGF: vascular endothelial growth factor, MMP: matrix metalloproteinase, c-Maf: cellular muscular aponeurotic fibrosarcoma, pSTAT1: phosphorylated signal transducer and activator of transcription 1, HIF: hypoxia inducible factor, vWF: von Willebrand factor)
